# Supplementary material for: Exploring the roles of men and masculinities in abortion and emergency contraception pathways, Ghana: a mobile phone-based mixed-methods study protocol
Source: BMJ Open. 2021 Feb 5;11(2):e042649. doi: 10.1136/bmjopen-2020-042649 (PMC7925914; doi:10.1136/bmjopen-2020-042649)
Supplement: Author's manuscript [file bmjopen-2020-042649.draft_revisions.pdf]

BMJ Open

BMJ Open is committed to open peer review. As part of this commitment we make the peer review history of every article we publish publicly available.

When an article is published we post the peer reviewers' comments and the authors' responses online. We also post the versions of the paper that were used during peer review. These are the versions that the peer review comments apply to.

The versions of the paper that follow are the versions that were submitted during the peer review process. They are not the versions of record or the final published versions. They should not be cited or distributed as the published version of this manuscript.

BMJ Open is an open access journal and the full, final, typeset and author-corrected version of record of the manuscript is available on our site with no access controls, subscription charges or pay-per-view fees (http://bmjopen.bmj.com).

If you have any questions on BMJ Open's open peer review process please email info.bmjopen@bmj.com

BMJ Open

Exploring the roles of men and masculinities in post-coital pregnancy avoidance, Ghana: a mobile phone-based mixed-methods study protocol

Journal:	*BMJ Open*
Manuscript ID	bmjopen-2020-042649
Article Type:	Protocol
Date Submitted by the Author:	13-Jul-2020
Complete List of Authors:	Strong, Joe; The London School of Economics and Political Science Department of Social Policy,
Keywords:	SEXUAL MEDICINE, PUBLIC HEALTH, QUALITATIVE RESEARCH, REPRODUCTIVE MEDICINE

SCHOLARONE™
Manuscripts

I, the Submitting Author has the right to grant and does grant on behalf of all authors of the Work (as defined in the below author licence), an exclusive licence and/or a non-exclusive licence for contributions from authors who are: i) UK Crown employees; ii) where BMJ has agreed a CC-BY licence shall apply, and/or iii) in accordance with the terms applicable for US Federal Government officers or employees acting as part of their official duties; on a worldwide, perpetual, irrevocable, royalty-free basis to BMJ Publishing Group Ltd ("BMJ") its licensees and where the relevant Journal is co-owned by BMJ to the co-owners of the Journal, to publish the Work in this journal and any other BMJ products and to exploit all rights, as set out in our licence.

The Submitting Author accepts and understands that any supply made under these terms is made by BMJ to the Submitting Author unless you are acting as an employee on behalf of your employer or a postgraduate student of an affiliated institution which is paying any applicable article publishing charge ("APC") for Open Access articles. Where the Submitting Author wishes to make the Work available on an Open Access basis (and intends to pay the relevant APC), the terms of reuse of such Open Access shall be governed by a Creative Commons licence – details of these licences and which Creative Commons licence will apply to this Work are set out in our licence referred to above.

Other than as permitted in any relevant BMJ Author's Self Archiving Policies, I confirm this Work has not been accepted for publication elsewhere, is not being considered for publication elsewhere and does not duplicate material already published. I confirm all authors consent to publication of this Work and authorise the granting of this licence.

For peer review only

Exploring the roles of men and masculinities in post-coital pregnancy avoidance, Ghana: a mobile phone-based mixed-methods study protocol

Joe Strong
j.strong3@lse.ac.uk
Department of Social Policy
London School of Economics and Political Science
Houghton Street
London
WC2A 2AE
UK

For peer review only

Word Count: 2824

Abstract

Introduction: Global commitments have established goals of achieving universal sexual and reproductive health and rights (SRHR) access, but critical obstacles remain. Emergency contraception and induced abortion – the most effective methods of post-coital pregnancy avoidance – are overlooked in policy and research. Men's roles in the SRHR of others are significant, particularly as obstacles to universal SRHR. Evidence on gender, masculinities and SRHR is essential to understand and reduce the barriers faced by individuals seeking post-coital pregnancy avoidance.

Methods and analysis: This study aims to understand men's masculinities and their relationships with emergency contraception and abortion. The protocol presents a multi-method study of men aged over 18 years in James Town, Accra, Ghana. In response to the COVID-19 pandemic, the research will use two mobile-based methods: a survey and in-depth interviews. Using respondent-driven sampling, an estimated 789 men will be recruited to participate in the survey, asking questions on their knowledge, attitude, behaviours and roles in emergency contraception and abortion. In-depth interviews focused on constructions of masculinity will be conducted with a purposive sample of men who participated in the survey. Data will be analysed concurrently using multiple regression analyses of quantitative data and abductive analysis of qualitative data.

Ethics and dissemination: Ethical approval has been granted by the London School of Economics and Political Science and the Ghana Health Service. The findings in this study will: engage with emerging research on masculinities and SRHR in Ghana and elsewhere; offer methodological insight for future research; and, provide evidence to inform interventions to reduce obstacles for emergency contraception and abortion care seekers. Dissemination will occur at all levels – policy, academic, community – including multiple academic articles, policy briefs, workshops and presentations, conference papers, and theatre/radio-based performances of key messages.

Keywords: Abortion, Emergency Contraception, SRHR, Men, Masculinity, Ghana

For peer review only

Article summary

Strengths and limitations of this study

- Transferring methods to mobile phones allows for research to be conducted in changing environments due to COVID-19, where movement and social contact are problematic.
- A mixed-methods approach strengthens the ability for the study to unpack critical constructions that relate to emergency contraception and induced abortion and increases the richness of the data.
- Mobile based methods are limiting in terms of only reaching those with access to the technology
- Mobile phones reduce non-verbal cues and lack of observable confirmation of privacy and consent do not allow for the inclusion of participants who have gatekeepers (e.g. young people)

For peer review only

For peer review only - http://bmjopen.bmj.com/site/about/guidelines.xhtml

INTRODUCTION

Post-coital pregnancy avoidance methods, the most effective of which are emergency contraception and induced abortions, are under-represented in global and regional policy and research.[1 2] Despite critical international milestones such as the International Conference on Population Development and Sustainable Development Goals 3 & 5[1] and regional commitments (e.g. Maputo Protocol[3]) calling for universal access to sexual and reproductive health and rights (SRHR), political, social and economic barriers have restricted the progress in SRHR, particularly in relation to post-coital pregnancy avoidance.[1 4-8]

Emergency contraception is most frequently provided in pill form. Though there is a copper IUD emergency contraceptive, evidence from high income countries indicates extremely low uptake.[9] There are various types of emergency contraception pills (ECPs) with varying efficacies – the most effective estimated to prevent up to 95% of pregnancies if taken within 5 days of sex that would lead to conception.[10] Abortions are essential healthcare, categorised by three differing levels of safety: safe, less safe, least safe.[11] The safety levels depend on the method choice and the environment where the abortion occurs. Methods to abort safely are dependent on gestational age and abortion type, including medication abortion, which can be used safely in non-clinical settings.[11]

Abortions and emergency contraception are both legally permissible in Ghana.[12 13] ECPs have been included in policy since 1996.[14] Knowledge of ECPs is increasing; the 2008 Ghana Demographic Health Survey, found knowledge among women at 35.4%.[15] More recently, PMA2020 estimated that 52% of women aged 15-49 had heard of emergency contraception.[16] ECPs are part of the mix of contraceptives that women in Ghana use,[17] with one third of young women attending pharmacies in Greater Accra reporting ECPs as their main method of contraception.[18]

Abortions have been decriminalised in Ghana since 1985 for three criteria: foetal abnormality; maternal health; rape / incest.[19] However, knowledge of the law remains low,[20] with only 11% of survey respondents knowing abortions were legally permitted.[21] Estimates of the abortion rate in Ghana vary from 13.4 per 1000 women aged 15-49 in 2017,[21] to 26.8 per 1000 in the same year.[20]

Globally, studies since the 1990s have shown that men have direct and indirect roles in emergency contraception[22-25] and abortion related care.[26-34] Research on emergency contraception and men's roles in Ghana has been limited.[17 18] Abortion-related studies in Ghana indicates that men's roles include exerting dominance via their role as partner or father,[35 36] controlling the finances for seeking care,[36] providing medicines and materials for care (e.g. procuring methods).[32 37] In addition, responses to (potential and confirmed) pregnancies,[38] including denial of pregnancy,[39] have a significant role in post-coital pregnancy avoidance decision making.

For peer review only - http://bmjopen.bmj.com/site/about/guidelines.xhtml

Few studies analyse the mechanisms that drive men's roles.[40 41] Critical studies of men and masculinities (CSM) has emerged as a response to this. CSM examines masculinities and femininities in order to understand men as gendered individuals.[42-44]

CSM draws heavily on the notion of 'hegemonic masculinity', which is the contextually idealised form of masculinity.[45 46] Hegemonic masculinities must be understood in the context of intersectional power relations, in which race, nationality, sexuality, ability all impact its construction.[47 48] Complex systems of power mean that masculinities might be hegemonic in one context but made marginal in another.[48-52] In Ghana, the destructive role of colonialism shaped masculinities by imposing Euro-American normative values, making existing masculinities marginal by forcing a white supremacist power system.[53] New forms of masculinities emerge and become embedded, defined in part by the exertion of power over others.[54 55]

Understanding better the characteristics of hegemonic and relational masculinities means that we can better understand men's roles in post-coital pregnancy avoidance. This understanding can promote change with the objective of ensuring universal access and autonomy for emergency contraception and abortion care users.

METHODS / DESIGN

Aims and Objectives

This protocol outlines a funded, multi-year research project, expected to finish in March 2023. The aim of this research is to generate extensive data exploring the relationships between masculinities and post-coital pregnancy avoidance in a study-site in Accra, Ghana. It has the following objectives:

1. To identify and explain the factors associated with men's knowledge, attitudes and behaviours towards post-coital pregnancy avoidance.
2. To generate new knowledge and analyse the interactions between men, masculinities and abortion pathways.
3. To investigate the significance of men and masculinities in the procurement and use of ECPs.

Data will be collected through a multi-method, concurrent design. A mixed methods approach is methodologically most appropriate to achieve these aims and objectives, allowing for both breadth of information through a larger survey, and depth of information through qualitative interviews.[56 57] These data will be used to produce substantial outputs, including peer-reviewed papers, blogs, policy briefs and art.

Study site

For peer review only - http://bmjopen.bmj.com/site/about/guidelines.xhtml

James Town was chosen as the study site. It is one of the smallest and most densely populated neighbourhoods in the Asheidu Keteke Sub-Metro of Accra. Access to healthcare services within James Town is very limited.[35 58] Pregnancy rates and estimated rates of less and least safe abortions are high.[35] The established relationship between gendered sexual norms and SRHR access[54] make it an appropriate case study site.

Formative research

This project has had two phases of formative research: a scoping trip in January 2019[59] and a piloting phase in February-March 2020. The formative research demonstrated that expert professionals emphasised the increasing use and importance of ECPs as a method of pregnancy avoidance in Ghana. The survey instrument was piloted (n=39) and iterated based on cognitive interview feedback from participants to ensure questions were understood consistently. The final instruments are available.[60]

Mobile Methods

Data collection will be conducted using mobile phones. The original research plan was to conduct the survey and interviews – alongside focus groups – face-to-face. However, the COVID-19 pandemic, declared 11 March 2020, necessitated a review of these methods. Mobile phones were the most appropriate option due to the high proportion – 94% - of shared and individual mobile phone ownership in urban Ghana.[61] The research team can be distanced from respondents and research can feasibly continue in the event of restricted movements.

Evidence suggests that telephone surveys are able to maintain similar quality standards to face-to-face interviews.[62] Interviews and surveys will be administered by the research team over the call. Self-administered surveys using smart technologies (e.g. mobile apps / web-based apps), and audio-visual platforms, were rejected, as they require a level of literacy and internet / data connectivity inappropriate for the context.[35]

Quantitative survey

Respondent Driven Sampling

Respondent driven sampling (RDS) was first conceptualised as an alternative to snowball sampling for hard to reach populations, for whom there was no reliable sampling frame to conduct a simple random sample.[63 64] As there are no appropriate existing mechanisms to identify men through social distanced, mobile phone means, RDS was considered suitable. It relies on a peer-referral system in which pre-existing relationships are utilised to create chains, with the intention being referrals from peer to peer eventually spread to the point that the final respondents are not known to the initial respondents.

Sample size

For peer review only - http://bmjopen.bmj.com/site/about/guidelines.xhtml

The sample of interest in this research are men aged 18 and over, who live in James Town. The survey asks respondents their gender in an open-ended format; this research includes anyone reporting that they are a man.

Estimating a sample size is important to provide a useful target minimum for the data to be statistically useful, although sample size estimation is difficult to do accurately for RDS.[65] The most frequently used method for calculating estimated sample size is to take the simple random sample size and multiple by a design effect ($deff$):[66]

$$n = deff \cdot \frac{P_A(1 - P_A)}{(se(\widehat{P_A}))^2}$$

P_A represents the proportion of the population of interest. This was taken as 0.73, which is the proportion of men aged 15-59 who believed that men should be involved in some aspect of SRHR.[67] The assumption is made that this holds true for a population of men aged 18+. The standard error (se) is set at 0.05.

Debate continues about an appropriate $deff$ value for RDS. The initial use of a $deff$ value of 2 has been shown to be too low for most RDS studies, and even a revised value of 4 might be lower than necessary.[68] To minimise the risk of having a $deff$ value too low for meaningful analysis, this survey assumes a $deff$ of 10, as used in recent RDS studies:[65]

$$n = 10 \cdot \frac{0.73(1 - 0.73)}{(0.05)^2} = 788.4$$

The study should reach an estimated 789 individuals to achieve a minimum estimated sample size.

Seeds and recruitment

Initially, RDS requires "seeds" who are ideally individuals with known networks within the sample of interest and become the first in the chain of peer-referral. The identification of seeds for this project will use the following matrix:

	Table 1: Sampling matrix for RDS seeds and interview respondents			
**Age Group**	James Town North		James Town South	
**18-25**	Ga	Non-Ga	Ga	Non-Ga
**26-39**	Ga	Non-Ga	Ga	Non-Ga

For peer review only

**40-59**	Ga	Non-Ga	Ga	Non-Ga
**60+**	Ga	Non-Ga	Ga	Non-Ga

The decision was made to stratify the seeds based on age, ethnicity (Ga/ non-Ga) and location (James Town North and James Town South) within the community in response to existing evidence[54] and observations and reflections from the research team of the most common factors affecting relationship networks.

Respondents will be compensated via mobile credit transfer of GHC 5[1] for responding and an additional GHC 2 per referral. Each respondent will be asked to find a maximum of 3 referrals. Respondents will be asked to provide potential referrals with the mobile number of the RA or provide the RA with the details to then call and arrange an interview time. Each respondent will be given a short code (said verbally and sent via SMS) to pass onto their referral, in order to track the peer-referral process.

Remote data checks

Mobile methods and the need for physical distancing mean that survey interviews cannot be checked in person by the Principle Investigator (PI)**.** A random respondent within each complete set of 10 surveys will be selected per RA to be data checked by a data assessor unaware of the original responses.

A selection of questions will be asked and surveys with inconsistencies in responses will be discarded and another survey randomly sampled for data checking. Re-interviewed respondents will receive additional compensation of GHC 3 for responding to the data check questions.

Data processing and analysis

Data will be processed onto RDSAT, a software designed specifically for RDS, to map networks and chains and to process the necessary weights. These weights will be identified by a series of questions in the survey that relate to the personal network size (PNS) of the individual.[65 69] The assumption is that the larger the network size, the greater likelihood of the respondent being selected to participate. Thus, weights are inversely proportional to the PNS.

[1] Ghana Cedi, currency in Ghana. GHC 1 : 1 USD. Exchange rate true on 24.06.20 per www.xe.com

For peer review only - http://bmjopen.bmj.com/site/about/guidelines.xhtml

The outcomes of interest are the factors associated with men's knowledge, attitudes and prior behaviour relating to post-coital pregnancy avoidance. Independent variables will include a composite socio-economic index, which combines questions on employment, residence type and resource availability. In addition, a composite score will be created based on responses to questions on the construction of masculinity. This novel approach will attempt to understand masculinities using quantitative data, which can subsequently be compared to qualitative findings.

Data will be cleaned and linear and odds regressions run to explore these associations between the outcomes of interest and the independent variables.

Qualitative sampling

In-depth interviews will be conducted from a sub-sample of the survey. This will be a non-probability sample, with respondents selected to populate the same matrix as that used for the seeds. The aim of this is to maximise obtaining a breadth of different experiences and responses. Sampling will be assumed to be complete once "saturation" is reached; when no new data are emerging from the selected sample.[57 70] Use of saturation means that a qualitative sample size cannot be pre-determined.

All survey respondents will be asked if they would be willing to participate in an interview at the end of the survey. The PI will select candidates purposively to populate the sample matrix (Table 1). Survey responses will be reviewed to ensure that diversity of individuals within each metric, until data are saturated. RAs will conduct the interviews and respondents will be compensated an additional GHC 10.

In-depth interview instruments

A semi-structured topic guide[60] will be used to explore conceptions of masculinities and elaborate on attitudes and behaviours around post-coital pregnancy avoidance. With consent, interviews will be recorded using devices that can plug into the RA's mobile phone, so that the audio is direct to the recorder and not audible for anyone but the interviewer, who will use headphones.

Interviews will be translated and transcribed. Transcribers will be hired with experience and fluency in Twi, Ga and English through an advertised, paid position in the study site. A random selection of 10% of interviews will be back translated by a separate Transcriber, to assess for quality. Significant difference in meaning will result in translations and transcriptions being redone by a third Transcriber. All documents will be stored on encrypted software and transferred through a secure platform between the PI and the RAs.

Qualitative analysis

For peer review only

Interviews are expected to generate a substantial and rich volume of data. As such, abductive analysis is appropriate, in which the analysis focuses on new and innovative findings, in order to test the evidence against existing theories of masculinities.[71] Emerging qualitative data will then be analysed concurrently with the outcomes from the survey. Qualitative data will be analysed using the RQDA platform, allowing integrated qualitative and quantitative data analysis on R.[72]

Participant and public involvement

Key experts were involved in the initial pilot phase of this study to elaborate on critical areas that could then be included in the development of the research question.[59] It was during this phase that emergency contraception was discussed as an important emerging contraceptive, warranting inclusion. Participants were included in the piloting phase of this study to provide feedback during surveys on the relevance of questions and to test the consistency of how questions are understood.

Local organisations provided support in the design phase, such as helping disseminate job advertisements and providing informal conversations about specific contextual factors relating to the research. This study intends to collaborate with local arts-based organisations to produce radio/theatre/other arts-based outputs for dissemination and engagement of the research findings.

DISCUSSION

Considerations and Limitations

There are methodological and practical considerations and limitations to this research. RDS assumes that respondents will randomly select referrals from their network, and that sub-populations are not isolated to the point of exclusion.[64] In reality, this is not likely to be the case, and whilst it is argued this is not necessarily a problem because skewed and nonsymmetric responses can be weighted accordingly,[64] it may impact the ability to make generalised population inferences.[65]

Individuals without access to mobile technology are likely to be missed, even if they have access to a shared mobile. Certain populations, such as adolescents and people with disabilities that impact their communication over technology, are excluded. This reflects the inability to utilise non-verbal cues to minimise the survey impact and to assess the role of a gatekeepers during the interview.

Ethics

Most important is the safety of the researchers and the respondents during the COVID-19 pandemic. This research will not require any face-to-face contact, nor any movement by RAs

For peer review only - http://bmjopen.bmj.com/site/about/guidelines.xhtml

For peer review only

that could expose them to unnecessary risk. A guaranteed salary ensures that payment will continue regardless of ability to collect data, until the end of the data collection period (estimated September 2020).

Informed consent will be sought verbally from all respondents and recorded by interviewers onto a document. All data will be stored securely on personal devices and transferred to the PI via secure networks, before being destroyed from any non-PI devices. Should respondents request more information, a fact sheet has been prepared with information on SRHR services and COVID-19-related care. Respondents will have the mobile number of the RAs in case of follow up questions in relation to the survey.

Ethical approval for this project has been granted by the London School of Economics and Political Science (REC ref. 000802c). Ethical approval for the amended research was granted from the Ghana Health Service Ethics Review Committee (GHS-ERC 008/11/19). Approval was also sought and obtained from the Ghana Health Services Regional Director for Greater Accra, and community stakeholders and leaders.

Dissemination

This research expects to produce a significant volume of rich data and diverse outputs. These data will be analysed and published into peer review papers, expected to be a minimum of three (per research aims and objectives) as well as additional papers reflecting on the methodological innovations.

Policy briefs will be created for dissemination amongst key stakeholders, particularly the Ghana Health Service. Community dissemination will include written and non-written mediums, in additional to partnering with local arts organisations to produce radio / theatre-based dissemination.

A multi-platform social media plan will be created to ensure necessary exposure and research uptake.

For peer review only

CONTRIBUTORSHIP STATEMENT

This research protocol was written in full by Joe Strong.

FUNDING

This work was supported by the Economic and Social Research Council as part of their Doctoral Training Programme at the London School of Economics and Political Science.

COMPETING INTERESTS

The author has no competing interests.

For peer review only - http://bmjopen.bmj.com/site/about/guidelines.xhtml

DATA SHARING STATEMENT

Data collected as part of this project will be available on UK Data Service and as part of the ESRC funding stipulations.

ACKNOWLEDGEMENTS

With thanks to Prof. Ernestina Coast and Dr Tiziana Leone for their support and comments on earlier drafts. Thanks to the Department of Social Policy, London School of Economics and Political Science and the Economic and Social Research Council for their support of this research project and to Katy Footman, Rornald Kananura, Dr Samantha R Lattof, Dr Rishita Nandagiri, Laura Sochas for their practical and methodological advice and support.

REFERENCES

1. Starrs AM, Ezeh AC, Barker G, et al. Accelerate progress - sexual and reproductive health and rights for all: report of the Guttmacher-Lancet Commission. *The Lancet* 2018;391(10140):2642-92. doi: 10.1016/S0140-6736(18)30293-9
2. Dover P. Gender and Development Cooperation: Scaling up Work with Men and Boys. *IDS Bulletin* 2014;45(1):91-98. doi: 10.1111/1759-5436.12073
3. Ahlberg BM, Kulane A. Sexual and reproductive health and rights. In: Tamale S, ed. African Sexualities: A Reader. Oxford, UK: Pambazuka Press 2011:313-39.
4. Farah F. Changing population paradigms post ICPD: policy and programme implications. *Genus* 2005;61:141-63. doi: 10.2307/29789272
5. World Health Organization. Strategy to Accelerate Progress towards the Attainment of International Development Goals and Targets Related to Reproductive Health. *Reproductive Health Matters* 2005;13(25):11-18. doi: 10.1016/S0968-8080(05)25166-2
6. Reddy B, Sen G. Breaking through the development silos: sexual and reproductive health and rights, Millennium Development Goals and gender equity - experiences from Mexico, India and Nigeria. *Reproductive Health Matters* 2013;21(42):18-31. doi: 10.1016/S0968-8080(13)42743-X
7. Yamin A, Boulanger V. Embedding sexual and reproductive health and rights in a transformational development framework: Lessons learned from the MDG targets and indicators. *Reproductive health matters* 2013;21:74-85. doi: 10.1016/S0968-8080(13)42727-1
8. Tamale S. Researching and theorising sexualities in Africa. In: Tamale S, ed. African Sexualities: A Reader. Oxford, UK: Pambazuka Press 2011:11-36.
9. Michie L, Cameron ST. Emergency contraception and impact on abortion rates. *Best Practice & Research Clinical Obstetrics & Gynaecology* 2020;63:111-19. doi: https://doi.org/10.1016/j.bpobgyn.2019.06.008
10. WHO. Emergency Contraception: Factsheet 2018 [Available from: https://www.who.int/news-room/fact-sheets/detail/emergency-contraception#:~:text=Emergency%20contraception%20(EC)%20can%20prevent,assault%20if%20without%20contraception%20coverage. accessed 25.06.20 2020.

For peer review only - http://bmjopen.bmj.com/site/about/guidelines.xhtml

11. Hook C, Miller A, Shand T, et al. Getting to Equal: Engaging Men and Boys in Sexual and Reproductive Health and Rights (SRHR) and Gender Equality. 2018
12. Kwankye SO, Cofie E. Ghana's Population Policy Implementation: Past, Present and Future. *2015* 2015;29(2) doi: 10.11564/29-2-738 [published Online First: 2015-09-23]
13. Baiden F, Awini E, Clerk C. Perception of university students in Ghana about emergency contraception. *Contraception* 2002;66(1):23-26. doi: https://doi.org/10.1016/S0010-7824(02)00315-3
14. Steiner MJ, Raymond E, Attafuah JD, et al. PROVIDER KNOWLEDGE ABOUT EMERGENCY CONTRACEPTION IN GHANA. *Journal of Biosocial Science* 2000;32(1):99-106. doi: 10.1017/S0021932000000997 [published Online First: 2000/01/01]
15. Ghana Statistical Service GSS, Ghana Health Service GHS, Macro ICF. Ghana Demographic and Health Survey 2008. Accra, Ghana: GSS, GHS, and ICF Macro, 2009.
16. ICEC. Knowledge and Use of Emergency Contraception in PMA2020 Surveys. New York: International Consortium for Emergency Contraception, 2017.
17. Engle KL, Hinson L, Chin-Quee D. "I love my ECPs": challenges to bridging emergency contraceptive users to more effective contraceptive methods in Ghana. *Journal of Family Planning and Reproductive Health Care* 2011;37(3):146. doi: 10.1136/jfprhc-2011-0077
18. Chin-Quee D, Hinson L, L'Engle KL, et al. Bridge over troubled waters: considerations in transitioning emergency contraceptive users to hormonal methods. *Contraception* 2012;85(4):363-68. doi: https://doi.org/10.1016/j.contraception.2011.07.014
19. Lithur NO. Destigmatising Abortion: Expanding Community Awareness of Abortion as a Reproductive Health Issue in Ghana. *African Journal of Reproductive Health / La Revue Africaine de la Sante; Reproductive* 2004;8(1):70-74. doi: 10.2307/3583308
20. Polis CB, Castillo PW, Otupiri E, et al. Estimating the incidence of abortion: using the Abortion Incidence Complications Methodology in Ghana, 2017. *BMJ Global Health* 2020;5(4):e002130. doi: 10.1136/bmjgh-2019-002130
21. Ghana Statistical Service GSS, Ghana Health Service GHS, Icf. Ghana Maternal Health Survey 2017. Accra, Ghana: GSS, GHS, and ICF, 2018.
22. Abiodun O. Use of emergency contraception in Nigeria: An exploration of related factors among sexually active female university students. *Sexual & Reproductive Healthcare* 2016;7:14-20. doi: https://doi.org/10.1016/j.srhc.2015.10.004
23. Marcell AV, Waks AB, Rutkow L, et al. What do we know about males and emergency contraception? A synthesis of the literature. *Perspect Sex Reprod Health* 2012;44(3):184-93. doi: 10.1363/4418412 [published Online First: 2012/09/11]
24. Daugherty JD. 'Should I Tell Him?' How Women Decide to Include or Exclude Their Partners in Emergency Contraception Decisions. *International Journal of Sexual Health* 2011;23(4):282-96. doi: 10.1080/19317611.2011.627090
25. Teixeira M, Guillaume A, Ferrand M, et al. Representations and uses of emergency contraception in West Africa. A social anthropological reading of a northern medicinal product. *Social Science & Medicine* 2012;75(1):148-55. doi: https://doi.org/10.1016/j.socscimed.2012.02.038
26. Coast E, Norris AH, Moore AM, et al. Trajectories of women's abortion-related care: A conceptual framework. *Social Science & Medicine* 2018;200:199-210. doi: https://doi.org/10.1016/j.socscimed.2018.01.035

For peer review only - http://bmjopen.bmj.com/site/about/guidelines.xhtml

27. Moore AM, Singh S, Bankole A. Do women and men consider abortion as an alternative to contraception in the United States? An exploratory study. *Glob Public Health* 2011;6 Suppl 1:S25-37. doi: 10.1080/17441692.2011.568948 [published Online First: 2011/05/19]
28. Moore AM, Jagwe-Wadda G, Bankole A. Men's attitudes about abortion in Uganda. *J Biosocial Science* 2011;43 doi: 10.1017/s0021932010000507
29. Moore AM, Bankole A, Awolude O, et al. Attitudes of women and men living with HIV and their healthcare providers towards pregnancy and abortion by HIV-positive women in Nigeria and Zambia. *African Journal of AIDS Research* 2015;14:29-42. doi: 10.2989/16085906.2015.1016981
30. Scott RH, Filippi V, Moore AM, et al. Setting the research agenda for induced abortion in Africa and Asia. *Int J Gynaecol Obstet* 2018;142(2):241-47. doi: 10.1002/ijgo.12525 [published Online First: 2018/05/11]
31. Sully EA, Madziyire MG, Riley T, et al. Abortion in Zimbabwe: A national study of the incidence of induced abortion, unintended pregnancy and post-abortion care in 2016. *PLOS ONE* 2018;13(10):e0205239. doi: 10.1371/journal.pone.0205239
32. Kumi-Kyereme A, Gbagbo FY, Amo-Adjei J. Role-players in abortion decision-making in the Accra Metropolis, Ghana. *Reprod Health* 2014;11:70. doi: 10.1186/1742-4755-11-70 [published Online First: 2014/09/18]
33. Freeman E, Coast E, Murray SF. Men's Roles in Women's Abortion Trajectories in Urban Zambia. *International Perspectives on Sexual and Reproductive Health* 2017;43(2):89-98. doi: 10.1363/43e4017
34. Coast E, Murray SF. "These things are dangerous": understanding induced abortion trajectories in urban Zambia. *Social Science & Medicine* 2016;153:201-09. doi: 10.1016/j.socscimed.2016.02.025
35. Bain LE, Zweekhorst MBM, Amoakoh-Coleman M, et al. To keep or not to keep? Decision making in adolescent pregnancies in Jamestown, Ghana. *PloS one* 2019;14(9):e0221789-e89. doi: 10.1371/journal.pone.0221789
36. Marlow HM, Awal AM, Antobam S, et al. Men's support for abortion in Upper East and Upper West Ghana. *Culture, Health & Sexuality* 2019:1-10. doi: 10.1080/13691058.2018.1545921
37. Rominski SD, Lori JR, Morhe ESK. "My friend who bought it for me, she has had an abortion before." The influence of Ghanaian women's social networks in determining the pathway to induced abortion. *Journal of Family Planning and Reproductive Health Care* 2017;43(3):216-21. doi: 10.1136/jfprhc-2016-101502
38. Challa S, Manu A, Morhe E, et al. Multiple levels of social influence on adolescent sexual and reproductive health decision-making and behaviors in Ghana. *Women & Health* 2018;58:434-50. doi: 10.1080/03630242.2017.1306607
39. Schwandt HM, Creanga AA, Adanu RM, et al. Pathways to unsafe abortion in Ghana: the role of male partners, women and health care providers. *Contraception* 2013;88 doi: 10.1016/j.contraception.2013.03.010
40. Greene ME, Biddlecom AE. Absent and Problematic Men: Demographic Accounts of Male Reproductive Roles. *Population and Development Review* 2000;26(1):81-115.
41. Ganle JK, Dery I. 'What men don't know can hurt women's health': a qualitative study of the barriers to and opportunities for men's involvement in maternal healthcare in Ghana. *Reproductive Health* 2015;12(93):(10 October 2015).
42. Law C. Men on the margins? Reflections on recruiting and engaging men in reproduction research. *Methodological Innovations* 2019;12(1):2059799119829425. doi: 10.1177/2059799119829425

For peer review only

43. Lohan M. How might we understand men's health better? Integrating explanations from critical studies on men and inequalities in health. *Soc Sci Med* 2007;65(3):493-504. doi: 10.1016/j.socscimed.2007.04.020 [published Online First: 2007/06/15]
44. Lohan M. Advancing Research on Men and Reproduction. *International Journal of Men's Health* 2015;14(3) doi: 10.3149/jmh.1403.214
45. Connell RW, Messerschmidt JW. Hegemonic Masculinity: Rethinking the Concept. *Gender & Society* 2005;19(6):829-59. doi: 10.1177/0891243205278639
46. Connell R. Masculinities. 2nd ed. ed. Berkeley, CA: Berkeley, CA : University of California Press 2005.
47. Demetriou DZ. Connell's concept of hegemonic masculinity: A critique. *Theory and Society* 2001;30(3):337-61. doi: 10.1023/A:1017596718715
48. Mfecane S. Towards African-centred theories of masculinity. *Social Dynamics* 2018;44(2):291-305. doi: 10.1080/02533952.2018.1481683
49. Miescher SF. Masculinities, Intersectionality, and Collaborative Approaches. *Men and Masculinities* 2008;11(2):227-33. doi: 10.1177/1097184X08315097
50. Chiweshe M. African men and feminism: Reflections on using African feminism in research. *Agenda* 2018;32(2):76-82. doi: 10.1080/10130950.2018.1460088
51. Ratele K. Currents against gender transformation of South African men: relocating marginality to the centre of research and theory of masculinities. *NORMA: International Journal for Masculinity Studies* 2014;9(1):30-44. doi: 10.1080/18902138.2014.892285
52. Ratele K. Hegemonic African Masculinities and Men's Heterosexual Lives: Some Uses for Homophobia. *African Studies Review* 2014;57(2):115-30. doi: 10.1017/asr.2014.50
53. Dery I, Apusigah AA. 'So Kuoɔ Kye Bɛ Yi': disrupting constructions of masculinities among the Dagaaba of Northwestern Ghana. *NORMA* 2020:1-17. doi: 10.1080/18902138.2020.1754014
54. Atobrah D. Men Play, Women Break the Town: Gender and Intergenerational Asymmetry in Sexual and Reproductive Worldview Among the Ga of Ghana. *Sexuality & Culture* 2017;21(3):860-82. doi: 10.1007/s12119-017-9426-x
55. Dery I, Ganle JK. "Who Knows, You May Overpower Him": Narratives and Experiences of Masculinities Among the Dagaaba Youth of Northwestern Ghana. *The Journal of Men's Studies* 2019;28(1):82-100. doi: 10.1177/1060826519846932
56. Onwuegbuzie AJ, Collins KM. A Typology of Mixed Methods Sampling Designs in Social Science Research. *The Qualitative Report* 2007;12(2):281-316.
57. Onwuegbuzie A, Leech N. Sampling Designs in Qualitative Research: Making the Sampling Process More Public2007.
58. Abu M, Codjoe SNA. Experience and Future Perceived Risk of Floods and Diarrheal Disease in Urban Poor Communities in Accra, Ghana. *International journal of environmental research and public health* 2018;15(12):2830. doi: 10.3390/ijerph15122830
59. Strong J. Reflections on Using a Scoping Trip as Part of a PhD Research Project in Sexual and Reproductive Health, Ghana. *SAGE Research Methods Cases* 2020 doi: 10.4135/9781529710724
60. Masculinities Project [Available from: https://www.masculinitiesproject.org/ accessed 29.06.2020 2020.
61. Zupork Dome M, Adu Duayeden K, Armah-Attoh D. Summary of Results, Ghana: Afrobarometer Round 8. Afrobarometer, 2020.

62. Irvine A. Duration, Dominance and Depth in Telephone and Face-to-Face Interviews: A Comparative Exploration. *International Journal of Qualitative Methods* 2011;10(3):202-20. doi: 10.1177/160940691101000302
63. Wejnert C, Pham H, Krishna N, et al. Estimating Design Effect and Calculating Sample Size for Respondent-Driven Sampling Studies of Injection Drug Users in the United States. *AIDS and Behavior* 2012;16(4):797-806. doi: 10.1007/s10461-012-0147-8
64. Volz E, Heckathorn D. Probability Based Estimation Theory for Respondent Driven Sampling. *Journal of Official Statistics* 2008;24:79-97.
65. Lattof SR. Collecting data from migrants in Ghana
Lessons learned using respondent-driven sampling. *Demographic Research* 2018;38:1017-58.
66. Salganik M. Variance Estimation, Design Effects, and Sample Size Calculations for Respondent-Driven Sampling. *Journal of urban health : bulletin of the New York Academy of Medicine* 2006;83:i98-112. doi: 10.1007/s11524-006-9106-x
67. Ghana Statistical Service GSS, Ghana Health Service GHS, International ICF. Ghana Demographic and Health Survey 2014. Rockville, Maryland, USA: GSS, GHS, and ICF International, 2015.
68. Johnston LG, Chen Y-H, Silva-Santisteban A, et al. An Empirical Examination of Respondent Driven Sampling Design Effects Among HIV Risk Groups from Studies Conducted Around the World. *AIDS and Behavior* 2013;17(6):2202-10. doi: 10.1007/s10461-012-0394-8
69. Decker MR, Marshall BD, Emerson M, et al. Respondent-driven sampling for an adolescent health study in vulnerable urban settings: a multi-country study. *The Journal of adolescent health : official publication of the Society for Adolescent Medicine* 2014;55(6 Suppl):S6-S12. doi: 10.1016/j.jadohealth.2014.07.021 [published Online First: 2014/11/19]
70. Onwuegbuzie AJ, Leech NL. Validity and Qualitative Research: An Oxymoron? *Quality & Quantity* 2007;41(2):233-49. doi: 10.1007/s11135-006-9000-3
71. Timmermans S, Tavory I. Theory Construction in Qualitative Research From Grounded Theory to Abductive Analysis. *Sociological Theory* 2012;30:167-86. doi: 10.1177/0735275112457914
72. What is RQDA and what are its features? [Available from: http://rqda.r-forge.r-project.org/ accessed 28.06.2020 2020.

For peer review only

For peer review only - http://bmjopen.bmj.com/site/about/guidelines.xhtml

BMJ Open

Exploring the roles of men and masculinities in abortion and emergency contraception pathways, Ghana: a mobile phone-based mixed-methods study protocol

Journal:	*BMJ Open*
Manuscript ID	bmjopen-2020-042649.R1
Article Type:	Protocol
Date Submitted by the Author:	22-Dec-2020
Complete List of Authors:	Strong, Joe; The London School of Economics and Political Science Department of Social Policy,
**Primary Subject Heading**:	Sexual health
Secondary Subject Heading:	Global health, Research methods
Keywords:	SEXUAL MEDICINE, PUBLIC HEALTH, QUALITATIVE RESEARCH, REPRODUCTIVE MEDICINE

SCHOLARONE™
Manuscripts

I, the Submitting Author has the right to grant and does grant on behalf of all authors of the Work (as defined in the below author licence), an exclusive licence and/or a non-exclusive licence for contributions from authors who are: i) UK Crown employees; ii) where BMJ has agreed a CC-BY licence shall apply, and/or iii) in accordance with the terms applicable for US Federal Government officers or employees acting as part of their official duties; on a worldwide, perpetual, irrevocable, royalty-free basis to BMJ Publishing Group Ltd ("BMJ") its licensees and where the relevant Journal is co-owned by BMJ to the co-owners of the Journal, to publish the Work in this journal and any other BMJ products and to exploit all rights, as set out in our licence.

The Submitting Author accepts and understands that any supply made under these terms is made by BMJ to the Submitting Author unless you are acting as an employee on behalf of your employer or a postgraduate student of an affiliated institution which is paying any applicable article publishing charge ("APC") for Open Access articles. Where the Submitting Author wishes to make the Work available on an Open Access basis (and intends to pay the relevant APC), the terms of reuse of such Open Access shall be governed by a Creative Commons licence – details of these licences and which Creative Commons licence will apply to this Work are set out in our licence referred to above.

Other than as permitted in any relevant BMJ Author's Self Archiving Policies, I confirm this Work has not been accepted for publication elsewhere, is not being considered for publication elsewhere and does not duplicate material already published. I confirm all authors consent to publication of this Work and authorise the granting of this licence.

For peer review only

Exploring the roles of men and masculinities in abortion and emergency contraception pathways, Ghana: a mobile phone-based mixed-methods study protocol

Joe Strong
j.strong3@lse.ac.uk
Department of Social Policy
London School of Economics and Political Science
Houghton Street
London
WC2A 2AE
UK

Word Count: 3996

For peer review only

Abstract

Introduction: Global commitments have established goals of achieving universal sexual and reproductive health and rights (SRHR) access, but critical obstacles remain. Emergency contraception and induced abortion are overlooked in policy and research. Men's roles in the SRHR of others are significant, particularly as obstacles to universal SRHR. Evidence on gender, masculinities and SRHR is essential to understand and reduce the barriers faced by individuals seeking to avoid the conception or continuation of a pregnancy.

Methods and analysis: This study aims to understand men's masculinities and their relationships with emergency contraception and abortion. The protocol presents a multi-method study of men aged over 18 years in James Town, Accra, Ghana. In response to the COVID-19 pandemic, the research will use two mobile-based methods: a survey and in-depth interviews. Using respondent-driven sampling, an estimated 789 men will be recruited to participate in the survey, asking questions on their knowledge, attitude, behaviours and roles in emergency contraception and abortion. In-depth interviews focused on constructions of masculinity will be conducted with a purposive sample of men who participated in the survey. Data will be analysed concurrently using multiple regression analyses of quantitative data and abductive analysis of qualitative data.

Ethics and dissemination: Ethical approval has been granted by the London School of Economics and Political Science and the Ghana Health Service. The findings in this study will: engage with emerging research on masculinities and SRHR in Ghana and elsewhere; offer methodological insight for future research; and provide evidence to inform interventions to reduce obstacles for emergency contraception and abortion care seekers. Dissemination will occur at all levels – policy, academic, community – including multiple academic articles, policy briefs, workshops and presentations, conference papers, and theatre/radio-based performances of key messages.

Keywords: Abortion, Emergency Contraception, SRHR, Men, Masculinity, Ghana

For peer review only

Article summary

Strengths and limitations of this study

- Transferring methods to mobile phones allows for research to be conducted in changing environments due to COVID-19, where movement and social contact are problematic.
- A mixed-methods approach strengthens the ability for the study to unpack critical constructions that relate to emergency contraception and induced abortion and increases the richness of the data.
- Mobile based methods are limiting in terms of only reaching those with access to the technology
- Mobile phones reduce non-verbal cues and lack of observable confirmation of privacy and consent do not allow for the inclusion of participants who have gatekeepers (e.g. young people)

For peer review only

INTRODUCTION

Post-coital pregnancy avoidance[1] methods, the most effective of which are emergency contraception and induced abortions, are under-represented in global and regional policy and research.[1 2] Despite critical international milestones such as the International Conference on Population Development and Sustainable Development Goals 3 & 5[1] and regional commitments (e.g. Maputo Protocol[3]) calling for universal access to sexual and reproductive health and rights (SRHR), political, social and economic barriers have restricted progress, particularly in relation to post-coital pregnancy avoidance.[1 4-8]

Emergency contraception is most frequently provided in pill form. Though there is a copper IUD emergency contraceptive, evidence from high income countries indicates extremely low uptake.[9] There are various types of emergency contraception pills (ECPs) with varying efficacies – the most effective estimated to prevent up to 95% of pregnancies if taken within 5 days of sex that would lead to conception.[10] Abortions are essential healthcare, categorised by the World Health Organisation (WHO) as safe, less safe, least safe.[11] The safety levels depend on the method choice and the environment where the abortion occurs. However, the advent of medical abortions – misoprostol / mifepristone combinations – has radically reshaped conceptions of safety, with pregnant people able to induce abortions safely outside of facilities, delineating the conflation of safety and over-medicalisation.[11 12]

Abortions and emergency contraception are both legally permissible in Ghana.[13 14] ECPs have been included in policy since 1996.[15] Knowledge of ECPs is increasing; the 2008 Ghana Demographic Health Survey found 35.4% of women had heard of emergency contraception when asked.[16] More recently, PMA2020 estimated that 52% of women aged 15-49 had heard of emergency contraception.[17] ECPs are part of the mix of contraceptives that women in Ghana use,[18] with one third of young women attending pharmacies in Greater Accra reporting ECPs as their main method of contraception.[19]

Abortions have been decriminalised in Ghana since 1985 for three criteria: foetal abnormality; maternal health; rape / incest.[20] However, knowledge of the law remains low,[21] with only 11% of survey respondents in the 2017 Ghana Maternal Health Survey knowing abortions were legally permitted.[22] Estimates of the abortion rate in Ghana vary from 13.4 per 1000 women aged 15-49 in 2017,[22] to 26.8 per 1000 in the same year,[21] many of which are less and least safe, with significant associated risks to pregnant people's health.[23 24]

[1] This is evolving terminology. It was important to indicate that this research would not be focused on methods of contraception that could be use pre or during intercourse (e.g. IUDs or condoms). Hence, these are methods that occur "post-coital", particularly post a sexual encounter that risks pregnancy. Emergency contraception and abortion are, however, separate but linked forms of reproductive healthcare. Emergency contraception prevents a pregnancy from occurring, whereas abortions are healthcare for people already pregnant. This distinction is important. "Avoidance" was used as an abbreviation of these methods that avoid either the conception or the continuation of a pregnancy.

Globally, studies since the 1990s have shown that men have direct and indirect roles in emergency contraception[25-28] and abortion related care.[29-37] Research on emergency contraception and men's roles in Ghana has been limited.[18 19] Abortion-related studies in Ghana indicate that men insert themselves in the autonomous decision-making of others via their role as partner or father,[38 39] including controlling the finances for seeking care,[39] and providing medicines and materials for care (e.g. procuring methods).[35 40] In addition, men's responses to (potential and confirmed) pregnancies,[41] including denial of pregnancy,[42] have a significant role in a person's post-coital pregnancy avoidance decision making. Whilst evidence indicates that men do support abortions, for example in instances of birth spacing and to finish school, such support continues to decentre pregnant people from their own, autonomous reproductive decision-making. [39]

Few studies analyse the mechanisms that drive men's roles reproduction.[43 44] Critical studies of men and masculinities (CSM) has emerged as a response to this. CSM examines masculinities and femininities in order to understand men as gendered individuals and provide an explanation of the mechanisms that drive social behaviours.[45-47] CSM draws heavily on the notion of 'hegemonic masculinity', which is the contextually idealised form of masculinity.[48 49] Hegemonic masculinities must be understood in the context of intersectional power relations, in which race, nationality, sexuality, ability all impact its construction.[50 51] Complex systems of power mean that masculinities might be hegemonic in one context but made marginal in another.[51-55] In Ghana, the destructive role of colonialism shaped masculinities by imposing Euro-American normative values, resulting in a complex system of masculinities embedded within a globalised white supremacist power system.[56]

Constructions of masculinities are not static, and new forms emerge and become intertwined in social structures – such as health and legal systems – and used to justify social control and dominance over other forms of masculinities and femininities.[57 58] Understanding masculinities is a mechanism through which to explore power and the potential ways in which these constructions operationalise power over reproductive decision-making. Investigating the characteristics of hegemonic and relational masculinities allows a better understanding of men's roles in post-coital pregnancy avoidance. As a constantly evolving construct, greater evidence on how masculinities operate presents the opportunity to consider how men's masculinities can be engaged with in policy and rights-based activism.

METHODS / DESIGN

Aims and Objectives

This protocol outlines a funded, multi-year research project, expected to finish in March 2022. The aim of this research is to generate extensive data exploring the relationships between masculinities and post-coital pregnancy avoidance in a study-site in Accra, Ghana. It has the following objectives:

For peer review only - http://bmjopen.bmj.com/site/about/guidelines.xhtml

1. To identify and explore the factors associated with men's knowledge, attitudes and behaviours towards post-coital pregnancy avoidance.
2. To generate and analyse new knowledge on how men's constructions of masculinities, sex and abortion interact with abortion-related care pathways.
3. To investigate the relationship between men and pathways to obtaining emergency contraception pills and to explore the constructions of masculinities within these.

Abortion and ECPs, whilst linked, are kept distinct in the objectives. This is to reflect the very different purposes each has within a person's reproductive life course, as well as variations in current knowledge and evidence about the two.

Data will be collected through a multi-method, concurrent design. A mixed methods approach is methodologically most appropriate to achieve these aims and objectives, allowing for both breadth of information through a larger survey, and depth of information through qualitative interviews.[59 60] These data will be used to produce substantial outputs, including peer-reviewed papers, blogs, policy briefs and art.

The qualitative and quantitative methods will be used in combination to explore all three objectives. Methods will be triangulated by looking at how masculinities are constructed and relate to abortions within each method and between each method, exposing complexities and narrative devices[61] that will allow for a generative exploration of the objectives.[62]

Formative research and Study Site Selection

This project had two phases of formative research: a scoping trip in January 2019 and a piloting phase in February-March 2020. The formative phase involved conversations with expert professionals working in SRHR, particularly abortion, including activists, advocates, academics and practitioners.[63] Such conversations resulted in the inclusion of ECPs into the research project. An important component of the scoping trip was to identify a relevant area to locate the study.

James Town, a neighbourhood in Accra, was chosen as the study site. Expert conversations indicated that it would be an area where the research topic could be useful for local activists as well as the Ghana Health Service and key stakeholders. Key stakeholders in the community, including the Secretary to the Paramount, a key figurehead in the Ga community in James Town, expressed approval for the research to be conducted.

The majority of residents are ethnically Ga, with largely internal migration meaning that there is an increasing mix of cultural representation from across Ghana.[64] Ga communities have been historically patrilineal, compounded by the imposition of patriarchal British values during colonialism. While relations between genders afford women certain autonomies, pregnancy and childbirth remain important and are linked to men's performance of masculinities.[57] The changing dynamics of the area, for example from economic internal

For peer review only - http://bmjopen.bmj.com/site/about/guidelines.xhtml

migration from elsewhere in Ghana,[64] allows for explorations of how constructions of masculinities might vary within and between groups of men.

Access to healthcare remains inequitable, with the Accra Metropolitan Assembly reporting "inadequate coverage" across of the entire sub-metro.[65] This includes challenges in both accessing healthcare and in public health, with recent incidences of diarrheal disease[66] and cholera[67] outbreaks. Despite a number of maternity-based clinics in James Town,[68] the largest of which is Ussher Polyclinic, it was observable that abortion services are not advertised. Evidence indicates that rates of less and least safe abortions remain high, with pregnant people preferring to self-manage their abortions outside of facilities, often with less safe methods such as herbs, toxins and pharmaceuticals.[38] In addition, the study indicated that partners, predominantly men, created barriers to abortion-related care through withholding moral support or financial resources.[38]

Prior to the second formative research phase, three research assistants (RAs) were hired. The researcher position was advertised on social media and through WhatsApp, with a selection of applicants interviewed. Following a week-long paid training workshop on methods and value-clarification on SRHR, the three researchers were asked to join the team. All are from the research context and the research tools were iteratively developed as a team.

During the piloting stage, 39 men aged 18 and above were purposively sampled from Ussher Town, a neighbourhood next to James Town. This aimed to reduce the likelihood of these men being resampled during the data collection phase. The survey instrument was iterated based on cognitive interview feedback from participants to ensure questions were understood consistently. The survey and interview guides are available at the project website: https://www.masculinitiesproject.org/

Mobile Methods

Data collection will be conducted using mobile phones. The original research plan was to conduct the survey and interviews – alongside focus groups – face-to-face. However, the COVID-19 pandemic, declared 11 March 2020, necessitated a review of these methods. Mobile phones were the most appropriate option due to the high proportion – 94% - of shared and individual mobile phone ownership in urban Ghana.[69] The research team can be distanced from respondents and research can feasibly continue in the event of restricted movements.

Evidence suggests that mobile phone-based data collection can provide rich and quality responses, though can require more reflexivity of how the mode might shape the data.[70 71] Interviews and surveys will be administered by the research team over the call. Self-administered surveys using smart technologies (e.g. mobile apps / web-based apps), and audio-visual platforms, were rejected, as they require a level of literacy and internet / data connectivity inappropriate for the context.[38]

For peer review only - http://bmjopen.bmj.com/site/about/guidelines.xhtml

Quantitative survey

Respondent Driven Sampling

Respondent driven sampling (RDS) was first conceptualised as an alternative to snowball sampling for populations less likely to participate in research, for whom there was no reliable sampling frame to conduct a simple random sample.[72 73] As there are no appropriate existing mechanisms to identify men through social distanced, mobile phone means, RDS was considered suitable. It relies on a peer-referral system in which pre-existing relationships are utilised to create chains, with the intention being referrals from peer to peer eventually spread to the point that the final respondents are not known to the initial respondents.

The outcomes of interest are the factors associated with men's knowledge, attitudes and prior behaviour relating to post-coital pregnancy avoidance. Independent variables will include a composite socio-economic index, which combines questions on employment, residence type and resource availability. In addition, a composite score will be created based on responses to questions on the construction of masculinity. This novel approach will attempt to understand masculinities using quantitative data, which can subsequently be compared to qualitative findings.

Sample size

The sample of interest in this research are men aged 18 and over, who live in James Town. The survey asks respondents their gender in an open-ended format; this research includes anyone reporting that they are a man.

Estimating a sample size is important to provide a useful target minimum for the data to be statistically useful, although sample size estimation is difficult to do accurately for RDS.[74] The most frequently used method for calculating estimated sample size is to take the simple random sample size and multiple by a design effect ($deff$):[75]

$$n = deff \cdot \frac{P_A(1 - P_A)}{(se(\widehat{P_A}))^2}$$

P_A represents the proportion of the population of interest. This was taken as 0.73, which is the proportion of men aged 15-59 who believed that men should be involved in some aspect of SRHR.[76] The assumption is made that this holds true for a population of men aged 18+. The standard error (se) is set at 0.05.

Debate continues about an appropriate $deff$ value for RDS. The initial use of a $deff$ value of 2 has been shown to be too low for most RDS studies, and even a revised value of 4 might

For peer review only - http://bmjopen.bmj.com/site/about/guidelines.xhtml

be lower than necessary.[77] To minimise the risk of having a $deff$ value too low for meaningful analysis, this survey assumes a $deff$ of 10, as used in recent RDS studies:[74]

$$n = 10 \cdot \frac{0.73(1 - 0.73)}{(0.05)^2} = 788.4$$

The study should reach an estimated 789 individuals to achieve a minimum estimated sample size.

Seeds and recruitment

Initially, RDS requires "seeds" who are ideally individuals with known networks within the sample of interest and become the first in the chain of peer-referral. The identification of seeds for this project will use the following matrix:

Table 1: Sampling matrix for RDS seeds and interview respondents

Age Group	James Town North		James Town South	
**18-25**	Ga	Non-Ga	Ga	Non-Ga
**26-39**	Ga	Non-Ga	Ga	Non-Ga
**40-59**	Ga	Non-Ga	Ga	Non-Ga
**60+**	Ga	Non-Ga	Ga	Non-Ga

The decision was made to stratify the seeds based on age, ethnicity (Ga/ non-Ga) and location (James Town North and James Town South) within the community in response to existing evidence[57] and observations and reflections from the research team of the most common factors affecting relationship networks.

Respondents will be compensated via mobile credit transfer of GHC 5[2] for responding and an additional GHC 2 per referral. Each respondent will be asked to find a maximum of 3 referrals. Respondents will be asked to provide potential referrals with the mobile number of the research assistant (RA) or provide the RA with the details to then call and arrange an

[2] Ghana Cedi, currency in Ghana. GHC 1 : 1 USD. Exchange rate true on 24.06.20 per www.xe.com

For peer review only

interview time. Each respondent will be given a short code (said verbally and sent via SMS) to pass onto their referral, in order to track the peer-referral process.

Remote data checks

Mobile methods and the need for physical distancing mean that survey interviews cannot be checked in person by the Principal Investigator (PI). A random respondent within each complete set of 10 surveys will be selected per RA to be data checked by a data assessor unaware of the original responses.

A selection of questions will be asked to assess for inconsistencies in questions and to ensure that the respondent had been interviewed. Re-interviewed respondents will receive additional compensation of GHC 3 for responding to the data check questions.

Conceptualising men's masculinities

The survey consists of seven sections: socio-demographics, household and socioeconomics, relationships, emergency contraception, abortion, masculinity and the abridged Washington Group Questions.[78]

The masculinities section was the most complex to design and benefited from the collaborative approach to this research design.[49 79] Through conversations with men during the preparation phases, piloting the survey with men in similar neighbourhoods around James Town, and workshop discussions with research team, a list of factors that were frequently associated with masculinities were created. Men are asked whether they believe that these factors are important for a man their age, as well as asked open ended questions of what factors make a 'good' man and a 'good' woman. The language of these questions was tested during the formative stages to ensure consistent comprehension.

Data processing and analysis

Data will be processed onto RDSAT, a software designed specifically for RDS, to map networks and chains and to process the necessary weights. These weights will be identified by a series of questions in the survey that relate to the personal network size (PNS) of the individual.[74 80] The assumption is that the larger the network size, the greater likelihood of the respondent being selected to participate. Thus, weights are inversely proportional to the PNS.

Data will be cleaned and linear and logistic regressions run to explore these associations between the outcomes of interest and the independent variables.

Qualitative sampling

In-depth interviews will be conducted from a sub-sample of the survey. This will be a non-probability sample, with respondents selected to populate the same matrix as that used for the

For peer review only - http://bmjopen.bmj.com/site/about/guidelines.xhtml

seeds. The aim of this is to maximise obtaining a breadth of different experiences and responses. This will take place concurrently alongside the survey and, if necessary, will continue after the survey has completed, to allow for all respondents to be considered as part of the sample.

Sampling will be assumed to be complete once "saturation" is reached; when no new data are emerging from the selected sample.[60 81] This project understands saturation as when no new themes or codes emerge.[82] As this research aims to be generative and exploratory, aligning approximately with inductive research, such an approach is recommended.[82]

If reaching saturation requires a significant volume of surveys, there may be financial constraints, as well as time constraints. Predictive sample sizes are difficult to estimate, though research using inductive approaches to saturation suggest that much can be established in the earliest interviewers.[83] The budget for interviews is not expected to be spent before saturation is reached.

All survey respondents will be asked if they would be willing to participate in an interview at the end of the survey. The PI will select candidates purposively to populate the sample matrix (Table 1). Survey responses will be reviewed to ensure that diversity of individuals within each metric, until data are saturated. RAs will conduct the interviews and respondents will be compensated an additional GHC 10.

In-depth interview instruments

A semi-structured topic guide[84] will be used to explore conceptions of masculinities and elaborate on attitudes and behaviours around post-coital pregnancy avoidance. With consent, interviews will be recorded using devices that can plug into the RA's mobile phone, so that the audio is direct to the recorder and not audible for anyone but the interviewer, who will use headphones.

Interviews will be translated and transcribed. Transcribers will be hired with experience and fluency in Twi, Ga and English through an advertised, paid position in the study site. A random selection of 10% of interviews will be back translated by a separate Transcriber, to assess for quality. Significant difference in meaning will result in translations and transcriptions being redone by a third Transcriber. All documents will be stored on encrypted software and transferred through a secure platform between the PI and the RAs.

Qualitative analysis

Interviews are expected to generate a substantial and rich volume of data. As such, abductive analysis is appropriate, in which the analysis focuses on new and innovative findings, in order to test the evidence against existing theories of masculinities.[85] Emerging qualitative data will then be analysed concurrently with the outcomes from the survey. Qualitative data

For peer review only - http://bmjopen.bmj.com/site/about/guidelines.xhtml

will be analysed using the RQDA platform, allowing integrated qualitative and quantitative data analysis on R.[86]

Participant and public involvement

Key experts (activists, advocates, practitioners, academics working in the field of SRHR) were involved in the scoping phase of this study to elaborate on critical areas that could then be included in the development of the research question.[63] It was during this phase that emergency contraception was discussed as an important emerging contraceptive, warranting inclusion. Participants were included in the piloting phase of this study to provide feedback during surveys on the relevance of questions and to test the consistency of how questions are understood.

Local organisations provided support in the design phase, such as helping disseminate job advertisements and providing informal conversations about specific contextual factors relating to the research. This study intends to collaborate with local arts-based organisations to produce radio/theatre/other arts-based outputs for dissemination and engagement of the research findings.

DISCUSSION

Considerations and Limitations

There are methodological and practical considerations and limitations to this research. RDS assumes that respondents will randomly select referrals from their network, and that sub-populations are not isolated to the point of exclusion.[73] In reality, this is not likely to be the case, and whilst it is argued this is not necessarily a problem because skewed and nonsymmetric responses can be weighted accordingly,[73] it may impact the ability to make generalised population inferences.[74]

Individuals without access to mobile technology are likely to be missed, even if they have access to a shared mobile. Certain populations, such as adolescents and people with disabilities that impact their communication over technology, are excluded. This reflects the inability to utilise non-verbal cues to minimise the survey impact and to assess the role of a gatekeepers during the interview.

Evidence indicates that the role of the interviewer has a direct relationship with the quality of answers relating to abortion. Men in this project might wish to provide what they perceive to be socially desirable answers, which are difficult to mitigate. The pilot survey offered little indication that this would be significant, evidenced by the number of men who offered additional information pertaining to 'sensitive' questions on SRHR. It is important, however, that the role of the interview be central in all the data analysis.

For peer review only

Moreover, one of the benefits of mobile phone use is that there is increased confidentiality, as only the respondent can hear the questions,[87] and men have control over when they would like to call and can choose the location they call from. This provides an interesting opportunity to consider the potential balancing of power that mobile phone methods could provide.

Ethics

Most important is the safety of the researchers and the respondents during the COVID-19 pandemic. This research will not require any face-to-face contact, nor any movement by RAs that could expose them to unnecessary risk. A guaranteed salary ensures that payment will continue regardless of ability to collect data, until the end of the data collection period (estimated September 2020).

Informed consent will be sought verbally from all respondents and recorded by interviewers onto a document. All data will be stored securely on personal devices and transferred to the PI via secure networks, before being destroyed from any non-PI devices.

The topic could also elicit discomfort in triggering experiences relating to sexual and reproductive health. Information and phone numbers to specialist helplines (Marie Stopes Ghana) will be provided to participants, and all participants will be given the contact of the research team, who will be using specific mobile phones and sim cards bought solely for project use, and the GHS Secretariat on their informed consent sheets for any follow up necessary, relating to the research.

Ethical approval for this project has been granted by the London School of Economics and Political Science (REC ref. 000802c). Ethical approval for the amended research was granted from the Ghana Health Service Ethics Review Committee (GHS-ERC 008/11/19). Approval was also sought and obtained from the Ghana Health Services Regional Director for Greater Accra, and community stakeholders and leaders.

Dissemination

This research expects to produce a significant volume of rich data and diverse outputs. These data will be analysed and published into peer review papers, expected to be a minimum of three (per research aims and objectives) as well as additional papers reflecting on the methodological innovations.

Policy briefs will be created for dissemination amongst key stakeholders, particularly the Ghana Health Service. Community dissemination will include written and non-written mediums, in additional to partnering with local arts organisations to produce radio / theatre-based dissemination.

For peer review only

The research team already has extensive experience in these areas within James Town. As these outputs can be time consuming, this experience, alongside the direct involvement in data collection, will be beneficial and create a more efficient process conceptualising feasible outputs, reliant on the successful application of funds.

CONTRIBUTORSHIP STATEMENT

This research protocol was written in full by Joe Strong.

FUNDING

This work was supported by the Economic and Social Research Council [grant number ES/P000622/1].

COMPETING INTERESTS

The author has no competing interests.

DATA AVAILABILITY

Data sharing is not currently applicable to this protocol as no datasets have been generated. Data will be made available once the data collection and study have been completed and available on UK Data Service as part of the ESRC funding stipulations.

ACKNOWLEDGEMENTS

Thanks to Samuel Nii Lante Lamptey, Richard Nii Kwartei Owoo, and Nii Kwartelai Quartey and Act for Change for their support and work within Ghana. With thanks to Prof. Ernestina Coast and Dr Tiziana Leone for their support and comments on earlier drafts. Thanks to the Department of Social Policy, London School of Economics and Political Science and the Economic and Social Research Council for their support of this research project and to Katy Footman, Rornald Kananura, Dr Samantha R Lattof, Dr Rishita Nandagiri, Laura Sochas for their practical and methodological advice and support.

REFERENCES

1. Starrs AM, Ezeh AC, Barker G, et al. Accelerate progress - sexual and reproductive health and rights for all: report of the Guttmacher-Lancet Commission. *The Lancet* 2018;391(10140):2642-92. doi: 10.1016/S0140-6736(18)30293-9
2. Dover P. Gender and Development Cooperation: Scaling up Work with Men and Boys. *IDS Bulletin* 2014;45(1):91-98. doi: 10.1111/1759-5436.12073
3. Ahlberg BM, Kulane A. Sexual and reproductive health and rights. In: Tamale S, ed. African Sexualities: A Reader. Oxford, UK: Pambazuka Press 2011:313-39.

For peer review only

4. Farah F. Changing population paradigms post ICPD: policy and programme implications. *Genus* 2005;61:141-63. doi: 10.2307/29789272
5. World Health Organization. Strategy to Accelerate Progress towards the Attainment of International Development Goals and Targets Related to Reproductive Health. *Reproductive Health Matters* 2005;13(25):11-18. doi: 10.1016/S0968-8080(05)25166-2
6. Reddy B, Sen G. Breaking through the development silos: sexual and reproductive health and rights, Millennium Development Goals and gender equity - experiences from Mexico, India and Nigeria. *Reproductive Health Matters* 2013;21(42):18-31. doi: 10.1016/S0968-8080(13)42743-X
7. Yamin A, Boulanger V. Embedding sexual and reproductive health and rights in a transformational development framework: Lessons learned from the MDG targets and indicators. *Reproductive health matters* 2013;21:74-85. doi: 10.1016/S0968-8080(13)42727-1
8. Tamale S. Researching and theorising sexualities in Africa. In: Tamale S, ed. African Sexualities: A Reader. Oxford, UK: Pambazuka Press 2011:11-36.
9. Michie L, Cameron ST. Emergency contraception and impact on abortion rates. *Best Practice & Research Clinical Obstetrics & Gynaecology* 2020;63:111-19. doi: https://doi.org/10.1016/j.bpobgyn.2019.06.008
10. WHO. Emergency Contraception: Factsheet 2018 [Available from: https://www.who.int/news-room/fact-sheets/detail/emergency-contraception#:~:text=Emergency%20contraception%20(EC)%20can%20prevent,assault%20if%20without%20contraception%20coverage. accessed 25.06.20 2020.
11. Ganatra B, Gerdts C, Rossier C, et al. Global, regional, and subregional classification of abortions by safety, 2010–14: estimates from a Bayesian hierarchical model. *The Lancet* 2017;390(10110):2372-81. doi: 10.1016/S0140-6736(17)31794-4
12. Hook C, Miller A, Shand T, et al. Getting to Equal: Engaging Men and Boys in Sexual and Reproductive Health and Rights (SRHR) and Gender Equality. 2018
13. Kwankye SO, Cofie E. Ghana's Population Policy Implementation: Past, Present and Future. *2015* 2015;29(2) doi: 10.11564/29-2-738 [published Online First: 2015-09-23]
14. Baiden F, Awini E, Clerk C. Perception of university students in Ghana about emergency contraception. *Contraception* 2002;66(1):23-26. doi: https://doi.org/10.1016/S0010-7824(02)00315-3
15. Steiner MJ, Raymond E, Attafuah JD, et al. PROVIDER KNOWLEDGE ABOUT EMERGENCY CONTRACEPTION IN GHANA. *Journal of Biosocial Science* 2000;32(1):99-106. doi: 10.1017/S0021932000000997 [published Online First: 2000/01/01]
16. Ghana Statistical Service GSS, Ghana Health Service GHS, Macro ICF. Ghana Demographic and Health Survey 2008. Accra, Ghana: GSS, GHS, and ICF Macro, 2009.
17. ICEC. Knowledge and Use of Emergency
Contraception in PMA2020 Surveys. New York: International Consortium for Emergency Contraception, 2017.
18. Engle KL, Hinson L, Chin-Quee D. "I love my ECPs": challenges to bridging emergency contraceptive users to more effective contraceptive methods in Ghana. *Journal of Family Planning and Reproductive Health Care* 2011;37(3):146. doi: 10.1136/jfprhc-2011-0077

For peer review only

19. Chin-Quee D, Hinson L, L'Engle KL, et al. Bridge over troubled waters: considerations in transitioning emergency contraceptive users to hormonal methods. *Contraception* 2012;85(4):363-68. doi: https://doi.org/10.1016/j.contraception.2011.07.014
20. Lithur NO. Destigmatising Abortion: Expanding Community Awareness of Abortion as a Reproductive Health Issue in Ghana. *African Journal of Reproductive Health / La Revue Africaine de la Sante; Reproductive* 2004;8(1):70-74. doi: 10.2307/3583308
21. Polis CB, Castillo PW, Otupiri E, et al. Estimating the incidence of abortion: using the Abortion Incidence Complications Methodology in Ghana, 2017. *BMJ Global Health* 2020;5(4):e002130. doi: 10.1136/bmjgh-2019-002130
22. Ghana Statistical Service GSS, Ghana Health Service GHS, Icf. Ghana Maternal Health Survey 2017. Accra, Ghana: GSS, GHS, and ICF, 2018.
23. Sundaram A, Juarez F, Bankole A, et al. Factors Associated with Abortion-Seeking and Obtaining a Safe Abortion in Ghana. *Studies in Family Planning* 2012;43(4):273-86.
24. Appiah-Agyekum NN. Medical abortions among university students in Ghana: implications for reproductive health education and management. *Int J Womens Health* 2018;10:515-22. doi: 10.2147/ijwh.s160297
25. Abiodun O. Use of emergency contraception in Nigeria: An exploration of related factors among sexually active female university students. *Sexual & Reproductive Healthcare* 2016;7:14-20. doi: https://doi.org/10.1016/j.srhc.2015.10.004
26. Marcell AV, Waks AB, Rutkow L, et al. What do we know about males and emergency contraception? A synthesis of the literature. *Perspect Sex Reprod Health* 2012;44(3):184-93. doi: 10.1363/4418412 [published Online First: 2012/09/11]
27. Daugherty JD. 'Should I Tell Him?' How Women Decide to Include or Exclude Their Partners in Emergency Contraception Decisions. *International Journal of Sexual Health* 2011;23(4):282-96. doi: 10.1080/19317611.2011.627090
28. Teixeira M, Guillaume A, Ferrand M, et al. Representations and uses of emergency contraception in West Africa. A social anthropological reading of a northern medicinal product. *Social Science & Medicine* 2012;75(1):148-55. doi: https://doi.org/10.1016/j.socscimed.2012.02.038
29. Coast E, Norris AH, Moore AM, et al. Trajectories of women's abortion-related care: A conceptual framework. *Social Science & Medicine* 2018;200:199-210. doi: https://doi.org/10.1016/j.socscimed.2018.01.035
30. Moore AM, Singh S, Bankole A. Do women and men consider abortion as an alternative to contraception in the United States? An exploratory study. *Glob Public Health* 2011;6 Suppl 1:S25-37. doi: 10.1080/17441692.2011.568948 [published Online First: 2011/05/19]
31. Moore AM, Jagwe-Wadda G, Bankole A. Men's attitudes about abortion in Uganda. *J Biosocial Science* 2011;43 doi: 10.1017/s0021932010000507
32. Moore AM, Bankole A, Awolude O, et al. Attitudes of women and men living with HIV and their healthcare providers towards pregnancy and abortion by HIV-positive women in Nigeria and Zambia. *African Journal of AIDS Research* 2015;14:29-42. doi: 10.2989/16085906.2015.1016981
33. Scott RH, Filippi V, Moore AM, et al. Setting the research agenda for induced abortion in Africa and Asia. *Int J Gynaecol Obstet* 2018;142(2):241-47. doi: 10.1002/ijgo.12525 [published Online First: 2018/05/11]
34. Sully EA, Madziyire MG, Riley T, et al. Abortion in Zimbabwe: A national study of the incidence of induced abortion, unintended pregnancy and post-abortion care in 2016. *PLOS ONE* 2018;13(10):e0205239. doi: 10.1371/journal.pone.0205239

For peer review only

For peer review only - http://bmjopen.bmj.com/site/about/guidelines.xhtml

35. Kumi-Kyereme A, Gbagbo FY, Amo-Adjei J. Role-players in abortion decision-making in the Accra Metropolis, Ghana. *Reprod Health* 2014;11:70. doi: 10.1186/1742-4755-11-70 [published Online First: 2014/09/18]
36. Freeman E, Coast E, Murray SF. Men's Roles in Women's Abortion Trajectories in Urban Zambia. *International Perspectives on Sexual and Reproductive Health* 2017;43(2):89-98. doi: 10.1363/43e4017
37. Coast E, Murray SF. "These things are dangerous": understanding induced abortion trajectories in urban Zambia. *Social Science & Medicine* 2016;153:201-09. doi: 10.1016/j.socscimed.2016.02.025
38. Bain LE, Zweekhorst MBM, Amoakoh-Coleman M, et al. To keep or not to keep? Decision making in adolescent pregnancies in Jamestown, Ghana. *PloS one* 2019;14(9):e0221789-e89. doi: 10.1371/journal.pone.0221789
39. Marlow HM, Awal AM, Antobam S, et al. Men's support for abortion in Upper East and Upper West Ghana. *Culture, Health & Sexuality* 2019:1-10. doi: 10.1080/13691058.2018.1545921
40. Rominski SD, Lori JR, Morhe ESK. "My friend who bought it for me, she has had an abortion before." The influence of Ghanaian women's social networks in determining the pathway to induced abortion. *Journal of Family Planning and Reproductive Health Care* 2017;43(3):216-21. doi: 10.1136/jfprhc-2016-101502
41. Challa S, Manu A, Morhe E, et al. Multiple levels of social influence on adolescent sexual and reproductive health decision-making and behaviors in Ghana. *Women & Health* 2018;58:434-50. doi: 10.1080/03630242.2017.1306607
42. Schwandt HM, Creanga AA, Adanu RM, et al. Pathways to unsafe abortion in Ghana: the role of male partners, women and health care providers. *Contraception* 2013;88 doi: 10.1016/j.contraception.2013.03.010
43. Greene ME, Biddlecom AE. Absent and Problematic Men: Demographic Accounts of Male Reproductive Roles. *Population and Development Review* 2000;26(1):81-115.
44. Ganle JK, Dery I. 'What men don't know can hurt women's health': a qualitative study of the barriers to and opportunities for men's involvement in maternal healthcare in Ghana. *Reproductive Health* 2015;12(93):(10 October 2015).
45. Law C. Men on the margins? Reflections on recruiting and engaging men in reproduction research. *Methodological Innovations* 2019;12(1):2059799119829425. doi: 10.1177/2059799119829425
46. Lohan M. How might we understand men's health better? Integrating explanations from critical studies on men and inequalities in health. *Social science & medicine (1982)* 2007;65(3):493-504. doi: 10.1016/j.socscimed.2007.04.020 [published Online First: 2007/06/15]
47. Lohan M. Advancing Research on Men and Reproduction. *International Journal of Men's Health* 2015;14(3) doi: 10.3149/jmh.1403.214
48. Connell RW, Messerschmidt JW. Hegemonic Masculinity: Rethinking the Concept. *Gender & Society* 2005;19(6):829-59. doi: 10.1177/0891243205278639
49. Connell R. Masculinities. 2nd ed. ed. Berkeley, CA: Berkeley, CA : University of California Press 2005.
50. Demetriou DZ. Connell's concept of hegemonic masculinity: A critique. *Theory and Society* 2001;30(3):337-61. doi: 10.1023/A:1017596718715
51. Mfecane S. Towards African-centred theories of masculinity. *Social Dynamics* 2018;44(2):291-305. doi: 10.1080/02533952.2018.1481683
52. Miescher SF. Masculinities, Intersectionality, and Collaborative Approaches. *Men and Masculinities* 2008;11(2):227-33. doi: 10.1177/1097184X08315097

For peer review only - http://bmjopen.bmj.com/site/about/guidelines.xhtml

53. Chiweshe M. African men and feminism: Reflections on using African feminism in research. *Agenda* 2018;32(2):76-82. doi: 10.1080/10130950.2018.1460088
54. Ratele K. Currents against gender transformation of South African men: relocating marginality to the centre of research and theory of masculinities. *NORMA: International Journal for Masculinity Studies* 2014;9(1):30-44. doi: 10.1080/18902138.2014.892285
55. Ratele K. Hegemonic African Masculinities and Men's Heterosexual Lives: Some Uses for Homophobia. *African Studies Review* 2014;57(2):115-30. doi: 10.1017/asr.2014.50
56. Dery I, Apusigah AA. 'So Kuoɔ Kye Bɛ Yi': disrupting constructions of masculinities among the Dagaaba of Northwestern Ghana. *NORMA* 2020:1-17. doi: 10.1080/18902138.2020.1754014
57. Atobrah D. Men Play, Women Break the Town: Gender and Intergenerational Asymmetry in Sexual and Reproductive Worldview Among the Ga of Ghana. *Sexuality & Culture* 2017;21(3):860-82. doi: 10.1007/s12119-017-9426-x
58. Dery I, Ganle JK. "Who Knows, You May Overpower Him": Narratives and Experiences of Masculinities Among the Dagaaba Youth of Northwestern Ghana. *The Journal of Men's Studies* 2019;28(1):82-100. doi: 10.1177/1060826519846932
59. Onwuegbuzie AJ, Collins KM. A Typology of Mixed Methods Sampling Designs in Social Science Research. *The Qualitative Report* 2007;12(2):281-316.
60. Onwuegbuzie A, Leech N. Sampling Designs in Qualitative Research: Making the Sampling Process More Public2007.
61. Cornwell J. Hard earned lives : accounts of health and illness from East London. London: London : Tavistock 1984.
62. Flick U. Doing Triangulation and Mixed Methods. 55 City Road, London, 2018.
63. Strong J. Reflections on Using a Scoping Trip as Part of a PhD Research Project in Sexual and Reproductive Health, Ghana. *SAGE Research Methods Cases* 2020 doi: 10.4135/9781529710724
64. Tutu R, Boateng J, Busingye J, et al. Asymmetry in an uneven place: migrants' lifestyles, social capital, and self-rated health status in James Town, Accra. *GeoJournal* 2017;82(5):907-21. doi: 10.1007/s10708-016-9723-1
65. AMA. 2016 Annual Progress Report: Ghana Shared Growth and Developent Agenda II 2017.
66. Abu M, Codjoe SNA. Experience and Future Perceived Risk of Floods and Diarrheal Disease in Urban Poor Communities in Accra, Ghana. *Int J Environ Res Public Health* 2018;15(12):2830. doi: 10.3390/ijerph15122830
67. Tutu RA, Gupta S, Elavarthi S, et al. Exploring the development of a household cholera-focused health literacy scale in James Town, Accra. *Journal of Infection and Public Health* 2019;12(1):62-69. doi: https://doi.org/10.1016/j.jiph.2018.08.006
68. Da Pilma Lekettey J, Dako-Gyeke P, Agyemang SA, et al. Alcohol consumption among pregnant women in James Town Community, Accra, Ghana. *Reproductive Health* 2017;14(1):<xocs:firstpage xmlns:xocs=""/>. doi: 10.1186/s12978-017-0384-4
69. Zupork Dome M, Adu Duayeden K, Armah-Attoh D. Summary of Results, Ghana: Afrobarometer Round 8. Afrobarometer, 2020.
70. Irvine A. Duration, Dominance and Depth in Telephone and Face-to-Face Interviews: A Comparative Exploration. *International Journal of Qualitative Methods* 2011;10(3):202-20. doi: 10.1177/160940691101000302
71. Holt A. Using the telephone for narrative interviewing: A research note. *Qualitative Research - QUAL RES* 2010;10:113-21. doi: 10.1177/1468794109348686

For peer review only

72. Wejnert C, Pham H, Krishna N, et al. Estimating Design Effect and Calculating Sample Size for Respondent-Driven Sampling Studies of Injection Drug Users in the United States. *AIDS and Behavior* 2012;16(4):797-806. doi: 10.1007/s10461-012-0147-8

73. Volz E, Heckathorn D. Probability Based Estimation Theory for Respondent Driven Sampling. *Journal of Official Statistics* 2008;24:79-97.

74. Lattof SR. Collecting data from migrants in Ghana
Lessons learned using respondent-driven sampling. *Demographic Research* 2018;38:1017-58.

75. Salganik M. Variance Estimation, Design Effects, and Sample Size Calculations for Respondent-Driven Sampling. *Journal of urban health : bulletin of the New York Academy of Medicine* 2006;83:i98-112. doi: 10.1007/s11524-006-9106-x

76. Ghana Statistical Service GSS, Ghana Health Service GHS, International ICF. Ghana Demographic and Health Survey 2014. Rockville, Maryland, USA: GSS, GHS, and ICF International, 2015.

77. Johnston LG, Chen Y-H, Silva-Santisteban A, et al. An Empirical Examination of Respondent Driven Sampling Design Effects Among HIV Risk Groups from Studies Conducted Around the World. *AIDS and Behavior* 2013;17(6):2202-10. doi: 10.1007/s10461-012-0394-8

78. Washington Group on Disability Statistics. Washington Group: Short set disability questions 2016 [Available from: http://www.washingtongroup-disability.com/washington-group-question-sets/short-set-of-disability-questions/ accessed 29.06.2020 2020.

79. Leung L, Miedema S, Warner X, et al. Making feminism count: integrating feminist research principles in large-scale quantitative research on violence against women and girls. *Gender & Development* 2019;27(3):427-47. doi: 10.1080/13552074.2019.1668142

80. Decker MR, Marshall BD, Emerson M, et al. Respondent-driven sampling for an adolescent health study in vulnerable urban settings: a multi-country study. *The Journal of adolescent health : official publication of the Society for Adolescent Medicine* 2014;55(6 Suppl):S6-S12. doi: 10.1016/j.jadohealth.2014.07.021 [published Online First: 2014/11/19]

81. Onwuegbuzie AJ, Leech NL. Validity and Qualitative Research: An Oxymoron? *Quality & Quantity* 2007;41(2):233-49. doi: 10.1007/s11135-006-9000-3

82. Saunders B, Sim J, Kingstone T, et al. Saturation in qualitative research: exploring its conceptualization and operationalization. *Quality & quantity* 2018;52(4):1893-907. doi: 10.1007/s11135-017-0574-8 [published Online First: 2017/09/14]

83. Hennink MM, Kaiser BN, Marconi VC. Code Saturation Versus Meaning Saturation: How Many Interviews Are Enough? *Qualitative Health Research* 2016;27(4):591-608. doi: 10.1177/1049732316665344

84. Masculinities Project [Available from: https://www.masculinitiesproject.org/ accessed 29.06.2020 2020.

85. Timmermans S, Tavory I. Theory Construction in Qualitative Research From Grounded Theory to Abductive Analysis. *Sociological Theory* 2012;30:167-86. doi: 10.1177/0735275112457914

86. What is RQDA and what are its features? [Available from: http://rqda.r-forge.r-project.org/ accessed 28.06.2020 2020.

87. Dillon B. Using mobile phones to collect panel data in developing countries. *Journal of International Development* 2012;24(4):518-27. doi: 10.1002/jid.1771

For peer review only - http://bmjopen.bmj.com/site/about/guidelines.xhtml

BMJ Open

Exploring the roles of men and masculinities in abortion and emergency contraception pathways, Ghana: a mobile phone-based mixed-methods study protocol

Journal:	*BMJ Open*
Manuscript ID	bmjopen-2020-042649.R2
Article Type:	Protocol
Date Submitted by the Author:	07-Jan-2021
Complete List of Authors:	Strong, Joe; The London School of Economics and Political Science Department of Social Policy,
**Primary Subject Heading**:	Sexual health
Secondary Subject Heading:	Global health, Research methods
Keywords:	SEXUAL MEDICINE, PUBLIC HEALTH, QUALITATIVE RESEARCH, REPRODUCTIVE MEDICINE

SCHOLARONE™
Manuscripts

I, the Submitting Author has the right to grant and does grant on behalf of all authors of the Work (as defined in the below author licence), an exclusive licence and/or a non-exclusive licence for contributions from authors who are: i) UK Crown employees; ii) where BMJ has agreed a CC-BY licence shall apply, and/or iii) in accordance with the terms applicable for US Federal Government officers or employees acting as part of their official duties; on a worldwide, perpetual, irrevocable, royalty-free basis to BMJ Publishing Group Ltd ("BMJ") its licensees and where the relevant Journal is co-owned by BMJ to the co-owners of the Journal, to publish the Work in this journal and any other BMJ products and to exploit all rights, as set out in our licence.

The Submitting Author accepts and understands that any supply made under these terms is made by BMJ to the Submitting Author unless you are acting as an employee on behalf of your employer or a postgraduate student of an affiliated institution which is paying any applicable article publishing charge ("APC") for Open Access articles. Where the Submitting Author wishes to make the Work available on an Open Access basis (and intends to pay the relevant APC), the terms of reuse of such Open Access shall be governed by a Creative Commons licence – details of these licences and which Creative Commons licence will apply to this Work are set out in our licence referred to above.

Other than as permitted in any relevant BMJ Author's Self Archiving Policies, I confirm this Work has not been accepted for publication elsewhere, is not being considered for publication elsewhere and does not duplicate material already published. I confirm all authors consent to publication of this Work and authorise the granting of this licence.

For peer review only

Exploring the roles of men and masculinities in abortion and emergency contraception pathways, Ghana: a mobile phone-based mixed-methods study protocol

Joe Strong
j.strong3@lse.ac.uk
Department of Social Policy
London School of Economics and Political Science
Houghton Street
London
WC2A 2AE
UK

Word Count: 4016

For peer review only

For peer review only - http://bmjopen.bmj.com/site/about/guidelines.xhtml

Abstract

Introduction: Global commitments have established goals of achieving universal sexual and reproductive health and rights (SRHR) access, but critical obstacles remain. Emergency contraception and induced abortion are overlooked in policy and research. Men's roles in the SRHR of others are significant, particularly as obstacles to universal SRHR. Evidence on gender, masculinities and SRHR is essential to understand and reduce the barriers faced by individuals seeking to avoid the conception or continuation of a pregnancy.

Methods and analysis: This study aims to understand men's masculinities and their relationships with emergency contraception and abortion. The protocol presents a multi-method study of men aged over 18 years in James Town, Accra, Ghana. In response to the COVID-19 pandemic, the research will use two mobile-based methods: a survey and in-depth interviews. Using respondent-driven sampling, an estimated 789 men will be recruited to participate in the survey, asking questions on their knowledge, attitude, behaviours and roles in emergency contraception and abortion. In-depth interviews focused on constructions of masculinity will be conducted with a purposive sample of men who participated in the survey. Data will be analysed concurrently using multiple regression analyses of quantitative data and abductive analysis of qualitative data.

Ethics and dissemination: Ethical approval has been granted by the London School of Economics and Political Science and the Ghana Health Service. The findings in this study will: engage with emerging research on masculinities and SRHR in Ghana and elsewhere; offer methodological insight for future research; and provide evidence to inform interventions to reduce obstacles for emergency contraception and abortion care seekers. Dissemination will occur at all levels – policy, academic, community – including multiple academic articles, policy briefs, workshops and presentations, conference papers, and theatre/radio-based performances of key messages.

Keywords: Abortion, Emergency Contraception, SRHR, Men, Masculinity, Ghana

For peer review only - http://bmjopen.bmj.com/site/about/guidelines.xhtml

Article summary

Strengths and limitations of this study

- Transferring methods to mobile phones allows for research to be conducted in changing environments due to COVID-19, where movement and social contact are problematic.
- A mixed-methods approach strengthens the ability for the study to unpack critical constructions that relate to emergency contraception and induced abortion and increases the richness of the data.
- Mobile based methods are limiting in terms of only reaching those with access to the technology
- Mobile phones reduce non-verbal cues and lack of observable confirmation of privacy and consent do not allow for the inclusion of participants who have gatekeepers (e.g. young people)

For peer review only

INTRODUCTION

Post-coital pregnancy avoidance[1] methods, the most effective of which are emergency contraception and induced abortions, are under-represented in global and regional policy and research.[1 2] Despite critical international milestones such as the International Conference on Population Development and Sustainable Development Goals 3 & 5[1] and regional commitments (e.g. Maputo Protocol[3]) calling for universal access to sexual and reproductive health and rights (SRHR), political, social and economic barriers have restricted progress, particularly in relation to post-coital pregnancy avoidance.[1 4-8]

Emergency contraception is most frequently provided in pill form. Though there is a copper IUD emergency contraceptive, evidence from high income countries indicates extremely low uptake.[9] There are various types of emergency contraception pills (ECPs) with varying efficacies – the most effective estimated to prevent up to 95% of pregnancies if taken within 5 days of sex that would lead to conception.[10] Abortions are essential healthcare, categorised by the World Health Organisation (WHO) as safe, less safe, least safe.[11] The safety levels depend on the method choice and the environment where the abortion occurs. However, the advent of medical abortions – misoprostol / mifepristone combinations – has radically reshaped conceptions of safety, with pregnant people able to induce abortions safely outside of facilities, delineating the conflation of safety and over-medicalisation.[11 12]

Abortions and emergency contraception are both legally permissible in Ghana.[13 14] ECPs have been included in policy since 1996.[15] Knowledge of ECPs is increasing; the 2008 Ghana Demographic Health Survey found 35.4% of women had heard of emergency contraception when asked.[16] More recently, PMA2020 estimated that 52% of women aged 15-49 had heard of emergency contraception.[17] ECPs are part of the mix of contraceptives that women in Ghana use,[18] with one third of young women attending pharmacies in Greater Accra reporting ECPs as their main method of contraception.[19]

Abortions have been decriminalised in Ghana since 1985 for three criteria: foetal abnormality; maternal health; rape / incest.[20] However, knowledge of the law remains low,[21] with only 11% of survey respondents in the 2017 Ghana Maternal Health Survey knowing abortions were legally permitted.[22] Estimates of the abortion rate in Ghana vary from 13.4 per 1000 women aged 15-49 in 2017,[22] to 26.8 per 1000 in the same year,[21] many of which are less and least safe, with significant associated risks to pregnant people's health.[23 24]

---

[1] This is evolving terminology. It was important to indicate that this research would not be focused on methods of contraception that could be use pre or during intercourse (e.g. IUDs or condoms). Hence, these are methods that occur "post-coital", particularly post a sexual encounter that risks pregnancy. Emergency contraception and abortion are, however, separate but linked forms of reproductive healthcare. Emergency contraception prevents a pregnancy from occurring, whereas abortions are healthcare for people already pregnant. This distinction is important. "Avoidance" was used as an abbreviation of these methods that avoid either the conception or the continuation of a pregnancy.

For peer review only

Globally, studies since the 1990s have shown that men have direct and indirect roles in emergency contraception[25-28] and abortion related care.[29-37] Research on emergency contraception and men's roles in Ghana has been limited.[18 19] Abortion-related studies in Ghana indicate that men insert themselves in the autonomous decision-making of others via their role as partner or father,[38 39] including controlling the finances for seeking care,[39] and providing medicines and materials for care (e.g. procuring methods).[35 40] In addition, men's responses to (potential and confirmed) pregnancies,[41] including denial of pregnancy,[42] have a significant role in a person's post-coital pregnancy avoidance decision making. Whilst evidence indicates that men do support abortions, for example in instances of birth spacing and to finish school, such support continues to decentre pregnant people from their own, autonomous reproductive decision-making. [39]

Few studies analyse the mechanisms that drive men's roles reproduction.[43 44] Critical studies of men and masculinities (CSM) has emerged as a response to this. CSM examines masculinities and femininities in order to understand men as gendered individuals and provide an explanation of the mechanisms that drive social behaviours.[45-47] CSM draws heavily on the notion of 'hegemonic masculinity', which is the contextually idealised form of masculinity.[48 49] Hegemonic masculinities must be understood in the context of intersectional power relations, in which race, nationality, sexuality, ability all impact its construction.[50 51] Complex systems of power mean that masculinities might be hegemonic in one context but made marginal in another.[51-55] In Ghana, the destructive role of colonialism shaped masculinities by imposing Euro-American normative values, resulting in a complex system of masculinities embedded within a globalised white supremacist power system.[56]

Constructions of masculinities are not static, and new forms emerge and become intertwined in social structures – such as health and legal systems – and used to justify social control and dominance over other forms of masculinities and femininities.[57 58] Understanding masculinities is a mechanism through which to explore power and the potential ways in which these constructions operationalise power over reproductive decision-making. Investigating the characteristics of hegemonic and relational masculinities allows a better understanding of men's roles in post-coital pregnancy avoidance. As a constantly evolving construct, greater evidence on how masculinities operate presents the opportunity to consider how men's masculinities can be engaged with in policy and rights-based activism.

METHODS / DESIGN

Aims and Objectives

This protocol outlines a funded, multi-year research project, expected to finish in March 2022. The aim of this research is to generate extensive data exploring the relationships between masculinities and post-coital pregnancy avoidance in a study-site in Accra, Ghana. It has the following objectives:

For peer review only

1. To identify and explore the factors associated with men's knowledge, attitudes and behaviours towards post-coital pregnancy avoidance.
2. To generate and analyse new knowledge on how men's constructions of masculinities, sex and abortion interact with abortion-related care pathways.
3. To investigate the relationship between men and pathways to obtaining emergency contraception pills and to explore the constructions of masculinities within these.

Abortion and ECPs, whilst linked, are kept distinct in the objectives. This is to reflect the very different purposes each has within a person's reproductive life course, as well as variations in current knowledge and evidence about the two.

Data will be collected through a multi-method, concurrent design. A mixed methods approach is methodologically most appropriate to achieve these aims and objectives, allowing for both breadth of information through a larger survey, and depth of information through qualitative interviews.[59 60] These data will be used to produce substantial outputs, including peer-reviewed papers, blogs, policy briefs and art.

The methods will be triangulated by looking at how masculinities are constructed and relate to abortions within each method and between each method, exposing complexities and narrative devices[61] that will allow for a generative exploration of the objectives.[62]

Objective 1: Quantitative data on sources of knowledge and the association between knowledge, attitudes and behaviours and socio-demographic factors will be analysed alongside an interrogation of qualitative data on the emergent mechanisms that drive these, with the intention of using uncovering broad trends and exploring the potential mechanisms behind these.

Objective 2 & 3: Interview data will explore the constructions and operationalisation of 'manhood' or 'masculinities' within discussions of sex, emergency contraception and abortion. Emergent themes will then be used to interrogate the responses men provide when asked about masculinities within the survey and to unpack any broader trends around the construction of masculinities and emergency contraception and abortion pathways, respectively. The data are designed to allow for an exploration of the links in surveys between men's involvement in emergency contraception and abortion pathways, their constructions of their masculinities, and the mechanisms emergent in qualitative data that might explain these.

Formative research and Study Site Selection

This project had two phases of formative research: a scoping trip in January 2019 and a piloting phase in February-March 2020. The formative phase involved conversations with expert professionals working in SRHR, particularly abortion, including activists, advocates, academics and practitioners.[63] Such conversations resulted in the inclusion of ECPs into the

For peer review only

research project. An important component of the scoping trip was to identify a relevant area to locate the study.

James Town, a neighbourhood in Accra, was chosen as the study site. Expert conversations indicated that it would be an area where the research topic could be useful for local activists as well as the Ghana Health Service and key stakeholders. Key stakeholders in the community, including the Secretary to the Paramount, a key figurehead in the Ga community in James Town, expressed approval for the research to be conducted.

The majority of residents are ethnically Ga, with largely internal migration meaning that there is an increasing mix of cultural representation from across Ghana.[64] Ga communities have been historically patrilineal, compounded by the imposition of patriarchal British values during colonialism. While relations between genders afford women certain autonomies, pregnancy and childbirth remain important and are linked to men's performance of masculinities.[57] The changing dynamics of the area, for example from economic internal migration from elsewhere in Ghana,[64] allows for explorations of how constructions of masculinities might vary within and between groups of men.

Access to healthcare remains inequitable, with the Accra Metropolitan Assembly reporting "inadequate coverage" across of the entire sub-metro.[65] This includes challenges in both accessing healthcare and in public health, with recent incidences of diarrheal disease[66] and cholera[67] outbreaks. Despite a number of maternity-based clinics in James Town,[68] the largest of which is Ussher Polyclinic, it was observable that abortion services are not advertised. Evidence indicates that rates of less and least safe abortions remain high, with pregnant people preferring to self-manage their abortions outside of facilities, often with less safe methods such as herbs, toxins and pharmaceuticals.[38] In addition, the study indicated that partners, predominantly men, created barriers to abortion-related care through withholding moral support or financial resources.[38]

Prior to the second formative research phase, three research assistants (RAs) were hired. The researcher position was advertised on social media and through WhatsApp, with a selection of applicants interviewed. Following a week-long paid training workshop on methods and value-clarification on SRHR, the three researchers were asked to join the team. All are from the research context and the research tools were iteratively developed as a team.

During the piloting stage, 39 men aged 18 and above were purposively sampled from Ussher Town, a neighbourhood next to James Town. This aimed to reduce the likelihood of these men being resampled during the data collection phase. The survey instrument was iterated based on cognitive interview feedback from participants to ensure questions were understood consistently. The survey and interview guides are available at the project website: https://www.masculinitiesproject.org/

Mobile Methods

For peer review only - http://bmjopen.bmj.com/site/about/guidelines.xhtml

Data collection will be conducted using mobile phones. The original research plan was to conduct the survey and interviews – alongside focus groups – face-to-face. However, the COVID-19 pandemic, declared 11 March 2020, necessitated a review of these methods. Mobile phones were the most appropriate option due to the high proportion – 94% - of shared and individual mobile phone ownership in urban Ghana.[69] The research team can be distanced from respondents and research can feasibly continue in the event of restricted movements.

Evidence suggests that mobile phone-based data collection can provide rich and quality responses, though can require more reflexivity of how the mode might shape the data.[70 71] Interviews and surveys will be administered by the research team over the call. Self-administered surveys using smart technologies (e.g. mobile apps / web-based apps), and audio-visual platforms, were rejected, as they require a level of literacy and internet / data connectivity inappropriate for the context.[38]

Quantitative survey

Respondent Driven Sampling

Respondent driven sampling (RDS) was first conceptualised as an alternative to snowball sampling for populations less likely to participate in research, for whom there was no reliable sampling frame to conduct a simple random sample.[72 73] As there are no appropriate existing mechanisms to identify men through social distanced, mobile phone means, RDS was considered suitable. It relies on a peer-referral system in which pre-existing relationships are utilised to create chains, with the intention being referrals from peer to peer eventually spread to the point that the final respondents are not known to the initial respondents.

The outcomes of interest are the factors associated with men's knowledge, attitudes and prior behaviour relating to post-coital pregnancy avoidance. Independent variables will include a composite socio-economic index, which combines questions on employment, residence type and resource availability. In addition, a composite score will be created based on responses to questions on the construction of masculinity. This novel approach will attempt to understand masculinities using quantitative data, which can subsequently be compared to qualitative findings.

Sample size

The sample of interest in this research are men aged 18 and over, who live in James Town. The survey asks respondents their gender in an open-ended format; this research includes anyone reporting that they are a man.

Estimating a sample size is important to provide a useful target minimum for the data to be statistically useful, although sample size estimation is difficult to do accurately for RDS.[74]

For peer review only - http://bmjopen.bmj.com/site/about/guidelines.xhtml

The most frequently used method for calculating estimated sample size is to take the simple random sample size and multiple by a design effect ($deff$):[75]

$$n = deff \cdot \frac{P_A(1 - P_A)}{(se(\widehat{P_A}))^2}$$

P_A represents the proportion of the population of interest. This was taken as 0.73, which is the proportion of men aged 15-59 who believed that men should be involved in some aspect of SRHR.[76] The assumption is made that this holds true for a population of men aged 18+. The standard error (se) is set at 0.05.

Debate continues about an appropriate $deff$ value for RDS. The initial use of a $deff$ value of 2 has been shown to be too low for most RDS studies, and even a revised value of 4 might be lower than necessary.[77] To minimise the risk of having a $deff$ value too low for meaningful analysis, this survey assumes a $deff$ of 10, as used in recent RDS studies:[74]

$$n = 10 \cdot \frac{0.73(1 - 0.73)}{(0.05)^2} = 788.4$$

The study should reach an estimated 789 individuals to achieve a minimum estimated sample size.

Seeds and recruitment

Initially, RDS requires “seeds” who are ideally individuals with known networks within the sample of interest and become the first in the chain of peer-referral. The identification of seeds for this project will use the following matrix:

Table 1: Sampling matrix for RDS seeds and interview respondents

Age Group	James Town North		James Town South	
18-25	Ga	Non-Ga	Ga	Non-Ga
26-39	Ga	Non-Ga	Ga	Non-Ga
40-59	Ga	Non-Ga	Ga	Non-Ga
60+	Ga	Non-Ga	Ga	Non-Ga

For peer review only - http://bmjopen.bmj.com/site/about/guidelines.xhtml

The decision was made to stratify the seeds based on age, ethnicity (Ga/ non-Ga) and location (James Town North and James Town South) within the community in response to existing evidence[57] and observations and reflections from the research team of the most common factors affecting relationship networks.

Respondents will be compensated via mobile credit transfer of GHC 5[2] for responding and an additional GHC 2 per referral. Each respondent will be asked to find a maximum of 3 referrals. Respondents will be asked to provide potential referrals with the mobile number of the research assistant (RA) or provide the RA with the details to then call and arrange an interview time. Each respondent will be given a short code (said verbally and sent via SMS) to pass onto their referral, in order to track the peer-referral process.

Remote data checks

Mobile methods and the need for physical distancing mean that survey interviews cannot be checked in person by the Principal Investigator (PI). A random respondent within each complete set of 10 surveys will be selected per RA to be data checked by a data assessor unaware of the original responses.

A selection of questions will be asked to assess for inconsistencies in questions and to ensure that the respondent had been interviewed. Re-interviewed respondents will receive additional compensation of GHC 3 for responding to the data check questions.

Conceptualising men's masculinities

The survey consists of seven sections: socio-demographics, household and socioeconomics, relationships, emergency contraception, abortion, masculinity and the abridged Washington Group Questions.[78]

The masculinities section was the most complex to design and benefited from the collaborative approach to this research design.[49 79] Through conversations with men during the preparation phases, piloting the survey with men in similar neighbourhoods around James Town, and workshop discussions with research team, a list of factors that were frequently associated with masculinities were created. Men are asked whether they believe that these factors are important for a man their age, as well as asked open ended questions of what factors make a 'good' man and a 'good' woman. The language of these questions was tested during the formative stages to ensure consistent comprehension.

Data processing and analysis

[2] Ghana Cedi, currency in Ghana. GHC 1 : 1 USD. Exchange rate true on 24.06.20 per www.xe.com

For peer review only - http://bmjopen.bmj.com/site/about/guidelines.xhtml

Data will be processed onto RDSAT, a software designed specifically for RDS, to map networks and chains and to process the necessary weights. These weights will be identified by a series of questions in the survey that relate to the personal network size (PNS) of the individual.[74 80] The assumption is that the larger the network size, the greater likelihood of the respondent being selected to participate. Thus, weights are inversely proportional to the PNS.

Data will be cleaned and linear and logistic regressions run to explore these associations between the outcomes of interest and the independent variables.

Qualitative sampling

In-depth interviews will be conducted from a sub-sample of the survey. This will be a non-probability sample, with respondents selected to populate the same matrix as that used for the seeds. The aim of this is to maximise obtaining a breadth of different experiences and responses. This will take place concurrently alongside the survey and, if necessary, will continue after the survey has completed, to allow for all respondents to be considered as part of the sample.

Sampling will be assumed to be complete once "saturation" is reached; when no new data are emerging from the selected sample.[60 81] This project understands saturation as when no new themes or codes emerge.[82] As this research aims to be generative and exploratory, aligning approximately with inductive research, such an approach is recommended.[82]

If reaching saturation requires a significant volume of surveys, there may be financial constraints, as well as time constraints. Predictive sample sizes are difficult to estimate, though research using inductive approaches to saturation suggest that much can be established in the earliest interviewers.[83] The budget for interviews is not expected to be spent before saturation is reached.

All survey respondents will be asked if they would be willing to participate in an interview at the end of the survey. The PI will select candidates purposively to populate the sample matrix (Table 1). Survey responses will be reviewed to ensure that diversity of individuals within each metric, until data are saturated. RAs will conduct the interviews and respondents will be compensated an additional GHC 10.

In-depth interview instruments

A semi-structured topic guide[84] will be used to explore conceptions of masculinities and elaborate on attitudes and behaviours around post-coital pregnancy avoidance. With consent, interviews will be recorded using devices that can plug into the RA's mobile phone, so that the audio is direct to the recorder and not audible for anyone but the interviewer, who will use headphones.

For peer review only - http://bmjopen.bmj.com/site/about/guidelines.xhtml

Interviews will be translated and transcribed. Transcribers will be hired with experience and fluency in Twi, Ga and English through an advertised, paid position in the study site. A random selection of 10% of interviews will be back translated by a separate Transcriber, to assess for quality. Significant difference in meaning will result in translations and transcriptions being redone by a third Transcriber. All documents will be stored on encrypted software and transferred through a secure platform between the PI and the RAs.

Qualitative analysis

Interviews are expected to generate a substantial and rich volume of data. As such, abductive analysis is appropriate, in which the analysis focuses on new and innovative findings, in order to test the evidence against existing theories of masculinities.[85] Emerging qualitative data will then be analysed concurrently with the outcomes from the survey. Qualitative data will be analysed using the RQDA platform, allowing integrated qualitative and quantitative data analysis on R.[86]

Participant and public involvement

Key experts (activists, advocates, practitioners, academics working in the field of SRHR) were involved in the scoping phase of this study to elaborate on critical areas that could then be included in the development of the research question.[63] It was during this phase that emergency contraception was discussed as an important emerging contraceptive, warranting inclusion. Participants were included in the piloting phase of this study to provide feedback during surveys on the relevance of questions and to test the consistency of how questions are understood.

Local organisations provided support in the design phase, such as helping disseminate job advertisements and providing informal conversations about specific contextual factors relating to the research. This study intends to collaborate with local arts-based organisations to produce radio/theatre/other arts-based outputs for dissemination and engagement of the research findings.

DISCUSSION

Considerations and Limitations

There are methodological and practical considerations and limitations to this research. RDS assumes that respondents will randomly select referrals from their network, and that sub-populations are not isolated to the point of exclusion.[73] In reality, this is not likely to be the case, and whilst it is argued this is not necessarily a problem because skewed and nonsymmetric responses can be weighted accordingly,[73] it may impact the ability to make generalised population inferences.[74]

For peer review only

For peer review only - http://bmjopen.bmj.com/site/about/guidelines.xhtml

Individuals without access to mobile technology are likely to be missed, even if they have access to a shared mobile. Certain populations, such as adolescents and people with disabilities that impact their communication over technology, are excluded. This reflects the inability to utilise non-verbal cues to minimise the survey impact and to assess the role of a gatekeepers during the interview.

Evidence indicates that the role of the interviewer has a direct relationship with the quality of answers relating to abortion. Men in this project might wish to provide what they perceive to be socially desirable answers, which are difficult to mitigate. The pilot survey offered little indication that this would be significant, evidenced by the number of men who offered additional information pertaining to 'sensitive' questions on SRHR. It is important, however, that the role of the interview be central in all the data analysis.

Moreover, one of the benefits of mobile phone use is that there is increased confidentiality, as only the respondent can hear the questions,[87] and men have control over when they would like to call and can choose the location they call from. This provides an interesting opportunity to consider the potential balancing of power that mobile phone methods could provide.

Ethics

Most important is the safety of the researchers and the respondents during the COVID-19 pandemic. This research will not require any face-to-face contact, nor any movement by RAs that could expose them to unnecessary risk. A guaranteed salary ensures that payment will continue regardless of ability to collect data, until the end of the data collection period (estimated September 2020).

Informed consent will be sought verbally from all respondents and recorded by interviewers onto a document. All data will be stored securely on personal devices and transferred to the PI via secure networks, before being destroyed from any non-PI devices.

The topic could also elicit discomfort in triggering experiences relating to sexual and reproductive health. Information and phone numbers to specialist helplines (Marie Stopes Ghana) will be provided to participants, and all participants will be given the contact of the research team, who will be using specific mobile phones and sim cards bought solely for project use, and the GHS Secretariat on their informed consent sheets for any follow up necessary, relating to the research.

Ethical approval for this project has been granted by the London School of Economics and Political Science (REC ref. 000802c). Ethical approval for the amended research was granted from the Ghana Health Service Ethics Review Committee (GHS-ERC 008/11/19). Approval was also sought and obtained from the Ghana Health Services Regional Director for Greater Accra, and community stakeholders and leaders.

For peer review only - http://bmjopen.bmj.com/site/about/guidelines.xhtml

Dissemination

This research expects to produce a significant volume of rich data and diverse outputs. These data will be analysed and published into peer review papers, expected to be a minimum of three (per research aims and objectives) as well as additional papers reflecting on the methodological innovations.

Policy briefs will be created for dissemination amongst key stakeholders, particularly the Ghana Health Service. Community dissemination will include written and non-written mediums, in additional to partnering with local arts organisations to produce radio / theatre-based dissemination.

The research team already has extensive experience in these areas within James Town. As these outputs can be time consuming, this experience, alongside the direct involvement in data collection, will be beneficial and create a more efficient process conceptualising feasible outputs, reliant on the successful application of funds.

CONTRIBUTORSHIP STATEMENT

This research protocol was written in full by Joe Strong.

FUNDING

This work was supported by the Economic and Social Research Council [grant number ES/P000622/1].

COMPETING INTERESTS

The author has no competing interests.

DATA AVAILABILITY

Data sharing is not currently applicable to this protocol as no datasets have been generated. Data will be made available once the data collection and study have been completed and available on UK Data Service as part of the ESRC funding stipulations.

ACKNOWLEDGEMENTS

Thanks to Samuel Nii Lante Lamptey, Richard Nii Kwartei Owoo, and Nii Kwartelai Quartey and Act for Change for their support and work within Ghana. With thanks to Prof. Ernestina Coast and Dr Tiziana Leone for their support and comments on earlier drafts. Thanks to the Department of Social Policy, London School of Economics and Political Science and the Economic and Social Research Council for their support of this research project and to Katy

For peer review only

Footman, Rornald Kananura, Dr Samantha R Lattof, Dr Rishita Nandagiri, Laura Sochas for their practical and methodological advice and support.

REFERENCES

1. Starrs AM, Ezeh AC, Barker G, et al. Accelerate progress - sexual and reproductive health and rights for all: report of the Guttmacher-Lancet Commission. *The Lancet* 2018;391(10140):2642-92. doi: 10.1016/S0140-6736(18)30293-9
2. Dover P. Gender and Development Cooperation: Scaling up Work with Men and Boys. *IDS Bulletin* 2014;45(1):91-98. doi: 10.1111/1759-5436.12073
3. Ahlberg BM, Kulane A. Sexual and reproductive health and rights. In: Tamale S, ed. African Sexualities: A Reader. Oxford, UK: Pambazuka Press 2011:313-39.
4. Farah F. Changing population paradigms post ICPD: policy and programme implications. *Genus* 2005;61:141-63. doi: 10.2307/29789272
5. World Health Organization. Strategy to Accelerate Progress towards the Attainment of International Development Goals and Targets Related to Reproductive Health. *Reproductive Health Matters* 2005;13(25):11-18. doi: 10.1016/S0968-8080(05)25166-2
6. Reddy B, Sen G. Breaking through the development silos: sexual and reproductive health and rights, Millennium Development Goals and gender equity - experiences from Mexico, India and Nigeria. *Reproductive Health Matters* 2013;21(42):18-31. doi: 10.1016/S0968-8080(13)42743-X
7. Yamin A, Boulanger V. Embedding sexual and reproductive health and rights in a transformational development framework: Lessons learned from the MDG targets and indicators. *Reproductive health matters* 2013;21:74-85. doi: 10.1016/S0968-8080(13)42727-1
8. Tamale S. Researching and theorising sexualities in Africa. In: Tamale S, ed. African Sexualities: A Reader. Oxford, UK: Pambazuka Press 2011:11-36.
9. Michie L, Cameron ST. Emergency contraception and impact on abortion rates. *Best Practice & Research Clinical Obstetrics & Gynaecology* 2020;63:111-19. doi: https://doi.org/10.1016/j.bpobgyn.2019.06.008
10. WHO. Emergency Contraception: Factsheet 2018 [Available from: https://www.who.int/news-room/fact-sheets/detail/emergency-contraception#:~:text=Emergency%20contraception%20(EC)%20can%20prevent,assault%20if%20without%20contraception%20coverage. accessed 25.06.20 2020.
11. Ganatra B, Gerdts C, Rossier C, et al. Global, regional, and subregional classification of abortions by safety, 2010–14: estimates from a Bayesian hierarchical model. *The Lancet* 2017;390(10110):2372-81. doi: 10.1016/S0140-6736(17)31794-4
12. Hook C, Miller A, Shand T, et al. Getting to Equal: Engaging Men and Boys in Sexual and Reproductive Health and Rights (SRHR) and Gender Equality. 2018
13. Kwankye SO, Cofie E. Ghana's Population Policy Implementation: Past, Present and Future. *2015* 2015;29(2) doi: 10.11564/29-2-738 [published Online First: 2015-09-23]
14. Baiden F, Awini E, Clerk C. Perception of university students in Ghana about emergency contraception. *Contraception* 2002;66(1):23-26. doi: https://doi.org/10.1016/S0010-7824(02)00315-3
15. Steiner MJ, Raymond E, Attafuah JD, et al. PROVIDER KNOWLEDGE ABOUT EMERGENCY CONTRACEPTION IN GHANA. *Journal of Biosocial Science*

For peer review only - http://bmjopen.bmj.com/site/about/guidelines.xhtml

2000;32(1):99-106. doi: 10.1017/S0021932000000997 [published Online First: 2000/01/01]
16. Ghana Statistical Service GSS, Ghana Health Service GHS, Macro ICF. Ghana Demographic and Health Survey 2008. Accra, Ghana: GSS, GHS, and ICF Macro, 2009.
17. ICEC. Knowledge and Use of Emergency
Contraception in PMA2020 Surveys. New York: International Consortium for Emergency Contraception, 2017.
18. Engle KL, Hinson L, Chin-Quee D. "I love my ECPs": challenges to bridging emergency contraceptive users to more effective contraceptive methods in Ghana. *Journal of Family Planning and Reproductive Health Care* 2011;37(3):146. doi: 10.1136/jfprhc-2011-0077
19. Chin-Quee D, Hinson L, L'Engle KL, et al. Bridge over troubled waters: considerations in transitioning emergency contraceptive users to hormonal methods. *Contraception* 2012;85(4):363-68. doi: https://doi.org/10.1016/j.contraception.2011.07.014
20. Lithur NO. Destigmatising Abortion: Expanding Community Awareness of Abortion as a Reproductive Health Issue in Ghana. *African Journal of Reproductive Health / La Revue Africaine de la Sante; Reproductive* 2004;8(1):70-74. doi: 10.2307/3583308
21. Polis CB, Castillo PW, Otupiri E, et al. Estimating the incidence of abortion: using the Abortion Incidence Complications Methodology in Ghana, 2017. *BMJ Global Health* 2020;5(4):e002130. doi: 10.1136/bmjgh-2019-002130
22. Ghana Statistical Service GSS, Ghana Health Service GHS, Icf. Ghana Maternal Health Survey 2017. Accra, Ghana: GSS, GHS, and ICF, 2018.
23. Sundaram A, Juarez F, Bankole A, et al. Factors Associated with Abortion-Seeking and Obtaining a Safe Abortion in Ghana. *Studies in Family Planning* 2012;43(4):273-86.
24. Appiah-Agyekum NN. Medical abortions among university students in Ghana: implications for reproductive health education and management. *Int J Womens Health* 2018;10:515-22. doi: 10.2147/ijwh.s160297
25. Abiodun O. Use of emergency contraception in Nigeria: An exploration of related factors among sexually active female university students. *Sexual & Reproductive Healthcare* 2016;7:14-20. doi: https://doi.org/10.1016/j.srhc.2015.10.004
26. Marcell AV, Waks AB, Rutkow L, et al. What do we know about males and emergency contraception? A synthesis of the literature. *Perspect Sex Reprod Health* 2012;44(3):184-93. doi: 10.1363/4418412 [published Online First: 2012/09/11]
27. Daugherty JD. 'Should I Tell Him?' How Women Decide to Include or Exclude Their Partners in Emergency Contraception Decisions. *International Journal of Sexual Health* 2011;23(4):282-96. doi: 10.1080/19317611.2011.627090
28. Teixeira M, Guillaume A, Ferrand M, et al. Representations and uses of emergency contraception in West Africa. A social anthropological reading of a northern medicinal product. *Social Science & Medicine* 2012;75(1):148-55. doi: https://doi.org/10.1016/j.socscimed.2012.02.038
29. Coast E, Norris AH, Moore AM, et al. Trajectories of women's abortion-related care: A conceptual framework. *Social Science & Medicine* 2018;200:199-210. doi: https://doi.org/10.1016/j.socscimed.2018.01.035
30. Moore AM, Singh S, Bankole A. Do women and men consider abortion as an alternative to contraception in the United States? An exploratory study. *Glob Public Health* 2011;6 Suppl 1:S25-37. doi: 10.1080/17441692.2011.568948 [published Online First: 2011/05/19]
31. Moore AM, Jagwe-Wadda G, Bankole A. Men's attitudes about abortion in Uganda. *J Biosocial Science* 2011;43 doi: 10.1017/s0021932010000507

For peer review only - http://bmjopen.bmj.com/site/about/guidelines.xhtml

32. Moore AM, Bankole A, Awolude O, et al. Attitudes of women and men living with HIV and their healthcare providers towards pregnancy and abortion by HIV-positive women in Nigeria and Zambia. *African Journal of AIDS Research* 2015;14:29-42. doi: 10.2989/16085906.2015.1016981
33. Scott RH, Filippi V, Moore AM, et al. Setting the research agenda for induced abortion in Africa and Asia. *Int J Gynaecol Obstet* 2018;142(2):241-47. doi: 10.1002/ijgo.12525 [published Online First: 2018/05/11]
34. Sully EA, Madziyire MG, Riley T, et al. Abortion in Zimbabwe: A national study of the incidence of induced abortion, unintended pregnancy and post-abortion care in 2016. *PLOS ONE* 2018;13(10):e0205239. doi: 10.1371/journal.pone.0205239
35. Kumi-Kyereme A, Gbagbo FY, Amo-Adjei J. Role-players in abortion decision-making in the Accra Metropolis, Ghana. *Reprod Health* 2014;11:70. doi: 10.1186/1742-4755-11-70 [published Online First: 2014/09/18]
36. Freeman E, Coast E, Murray SF. Men's Roles in Women's Abortion Trajectories in Urban Zambia. *International Perspectives on Sexual and Reproductive Health* 2017;43(2):89-98. doi: 10.1363/43e4017
37. Coast E, Murray SF. "These things are dangerous": understanding induced abortion trajectories in urban Zambia. *Social Science & Medicine* 2016;153:201-09. doi: 10.1016/j.socscimed.2016.02.025
38. Bain LE, Zweekhorst MBM, Amoakoh-Coleman M, et al. To keep or not to keep? Decision making in adolescent pregnancies in Jamestown, Ghana. *PloS one* 2019;14(9):e0221789-e89. doi: 10.1371/journal.pone.0221789
39. Marlow HM, Awal AM, Antobam S, et al. Men's support for abortion in Upper East and Upper West Ghana. *Culture, Health & Sexuality* 2019:1-10. doi: 10.1080/13691058.2018.1545921
40. Rominski SD, Lori JR, Morhe ESK. "My friend who bought it for me, she has had an abortion before." The influence of Ghanaian women's social networks in determining the pathway to induced abortion. *Journal of Family Planning and Reproductive Health Care* 2017;43(3):216-21. doi: 10.1136/jfprhc-2016-101502
41. Challa S, Manu A, Morhe E, et al. Multiple levels of social influence on adolescent sexual and reproductive health decision-making and behaviors in Ghana. *Women & Health* 2018;58:434-50. doi: 10.1080/03630242.2017.1306607
42. Schwandt HM, Creanga AA, Adanu RM, et al. Pathways to unsafe abortion in Ghana: the role of male partners, women and health care providers. *Contraception* 2013;88 doi: 10.1016/j.contraception.2013.03.010
43. Greene ME, Biddlecom AE. Absent and Problematic Men: Demographic Accounts of Male Reproductive Roles. *Population and Development Review* 2000;26(1):81-115.
44. Ganle JK, Dery I. 'What men don't know can hurt women's health': a qualitative study of the barriers to and opportunities for men's involvement in maternal healthcare in Ghana. *Reproductive Health* 2015;12(93):(10 October 2015).
45. Law C. Men on the margins? Reflections on recruiting and engaging men in reproduction research. *Methodological Innovations* 2019;12(1):2059799119829425. doi: 10.1177/2059799119829425
46. Lohan M. How might we understand men's health better? Integrating explanations from critical studies on men and inequalities in health. *Social science & medicine (1982)* 2007;65(3):493-504. doi: 10.1016/j.socscimed.2007.04.020 [published Online First: 2007/06/15]
47. Lohan M. Advancing Research on Men and Reproduction. *International Journal of Men's Health* 2015;14(3) doi: 10.3149/jmh.1403.214

For peer review only - http://bmjopen.bmj.com/site/about/guidelines.xhtml

48. Connell RW, Messerschmidt JW. Hegemonic Masculinity: Rethinking the Concept. *Gender & Society* 2005;19(6):829-59. doi: 10.1177/0891243205278639
49. Connell R. Masculinities. 2nd ed. ed. Berkeley, CA: Berkeley, CA : University of California Press 2005.
50. Demetriou DZ. Connell's concept of hegemonic masculinity: A critique. *Theory and Society* 2001;30(3):337-61. doi: 10.1023/A:1017596718715
51. Mfecane S. Towards African-centred theories of masculinity. *Social Dynamics* 2018;44(2):291-305. doi: 10.1080/02533952.2018.1481683
52. Miescher SF. Masculinities, Intersectionality, and Collaborative Approaches. *Men and Masculinities* 2008;11(2):227-33. doi: 10.1177/1097184X08315097
53. Chiweshe M. African men and feminism: Reflections on using African feminism in research. *Agenda* 2018;32(2):76-82. doi: 10.1080/10130950.2018.1460088
54. Ratele K. Currents against gender transformation of South African men: relocating marginality to the centre of research and theory of masculinities. *NORMA: International Journal for Masculinity Studies* 2014;9(1):30-44. doi: 10.1080/18902138.2014.892285
55. Ratele K. Hegemonic African Masculinities and Men's Heterosexual Lives: Some Uses for Homophobia. *African Studies Review* 2014;57(2):115-30. doi: 10.1017/asr.2014.50
56. Dery I, Apusigah AA. 'So Kuɔɔ Kye Bɛ Yi': disrupting constructions of masculinities among the Dagaaba of Northwestern Ghana. *NORMA* 2020:1-17. doi: 10.1080/18902138.2020.1754014
57. Atobrah D. Men Play, Women Break the Town: Gender and Intergenerational Asymmetry in Sexual and Reproductive Worldview Among the Ga of Ghana. *Sexuality & Culture* 2017;21(3):860-82. doi: 10.1007/s12119-017-9426-x
58. Dery I, Ganle JK. "Who Knows, You May Overpower Him": Narratives and Experiences of Masculinities Among the Dagaaba Youth of Northwestern Ghana. *The Journal of Men's Studies* 2019;28(1):82-100. doi: 10.1177/1060826519846932
59. Onwuegbuzie AJ, Collins KM. A Typology of Mixed Methods Sampling Designs in Social Science Research. *The Qualitative Report* 2007;12(2):281-316.
60. Onwuegbuzie A, Leech N. Sampling Designs in Qualitative Research: Making the Sampling Process More Public2007.
61. Cornwell J. Hard earned lives : accounts of health and illness from East London. London: London : Tavistock 1984.
62. Flick U. Doing Triangulation and Mixed Methods. 55 City Road, London, 2018.
63. Strong J. Reflections on Using a Scoping Trip as Part of a PhD Research Project in Sexual and Reproductive Health, Ghana. *SAGE Research Methods Cases* 2020 doi: 10.4135/9781529710724
64. Tutu R, Boateng J, Busingye J, et al. Asymmetry in an uneven place: migrants' lifestyles, social capital, and self-rated health status in James Town, Accra. *GeoJournal* 2017;82(5):907-21. doi: 10.1007/s10708-016-9723-1
65. AMA. 2016 Annual Progress Report: Ghana Shared Growth and Develodent Agenda II 2017.
66. Abu M, Codjoe SNA. Experience and Future Perceived Risk of Floods and Diarrheal Disease in Urban Poor Communities in Accra, Ghana. *Int J Environ Res Public Health* 2018;15(12):2830. doi: 10.3390/ijerph15122830
67. Tutu RA, Gupta S, Elavarthi S, et al. Exploring the development of a household cholera-focused health literacy scale in James Town, Accra. *Journal of Infection and Public Health* 2019;12(1):62-69. doi: https://doi.org/10.1016/j.jiph.2018.08.006

For peer review only

68. Da Pilma Lekettey J, Dako-Gyeke P, Agyemang SA, et al. Alcohol consumption among pregnant women in James Town Community, Accra, Ghana. *Reproductive Health* 2017;14(1):<xocs:firstpage xmlns:xocs=""/>. doi: 10.1186/s12978-017-0384-4

69. Zupork Dome M, Adu Duayeden K, Armah-Attoh D. Summary of Results, Ghana: Afrobarometer Round 8. Afrobarometer, 2020.

70. Irvine A. Duration, Dominance and Depth in Telephone and Face-to-Face Interviews: A Comparative Exploration. *International Journal of Qualitative Methods* 2011;10(3):202-20. doi: 10.1177/160940691101000302

71. Holt A. Using the telephone for narrative interviewing: A research note. *Qualitative Research - QUAL RES* 2010;10:113-21. doi: 10.1177/1468794109348686

72. Wejnert C, Pham H, Krishna N, et al. Estimating Design Effect and Calculating Sample Size for Respondent-Driven Sampling Studies of Injection Drug Users in the United States. *AIDS and Behavior* 2012;16(4):797-806. doi: 10.1007/s10461-012-0147-8

73. Volz E, Heckathorn D. Probability Based Estimation Theory for Respondent Driven Sampling. *Journal of Official Statistics* 2008;24:79-97.

74. Lattof SR. Collecting data from migrants in Ghana
Lessons learned using respondent-driven sampling. *Demographic Research* 2018;38:1017-58.

75. Salganik M. Variance Estimation, Design Effects, and Sample Size Calculations for Respondent-Driven Sampling. *Journal of urban health : bulletin of the New York Academy of Medicine* 2006;83:i98-112. doi: 10.1007/s11524-006-9106-x

76. Ghana Statistical Service GSS, Ghana Health Service GHS, International ICF. Ghana Demographic and Health Survey 2014. Rockville, Maryland, USA: GSS, GHS, and ICF International, 2015.

77. Johnston LG, Chen Y-H, Silva-Santisteban A, et al. An Empirical Examination of Respondent Driven Sampling Design Effects Among HIV Risk Groups from Studies Conducted Around the World. *AIDS and Behavior* 2013;17(6):2202-10. doi: 10.1007/s10461-012-0394-8

78. Washington Group on Disability Statistics. Washington Group: Short set disability questions 2016 [Available from: http://www.washingtongroup-disability.com/washington-group-question-sets/short-set-of-disability-questions/ accessed 29.06.2020 2020.

79. Leung L, Miedema S, Warner X, et al. Making feminism count: integrating feminist research principles in large-scale quantitative research on violence against women and girls. *Gender & Development* 2019;27(3):427-47. doi: 10.1080/13552074.2019.1668142

80. Decker MR, Marshall BD, Emerson M, et al. Respondent-driven sampling for an adolescent health study in vulnerable urban settings: a multi-country study. *The Journal of adolescent health : official publication of the Society for Adolescent Medicine* 2014;55(6 Suppl):S6-S12. doi: 10.1016/j.jadohealth.2014.07.021 [published Online First: 2014/11/19]

81. Onwuegbuzie AJ, Leech NL. Validity and Qualitative Research: An Oxymoron? *Quality & Quantity* 2007;41(2):233-49. doi: 10.1007/s11135-006-9000-3

82. Saunders B, Sim J, Kingstone T, et al. Saturation in qualitative research: exploring its conceptualization and operationalization. *Quality & quantity* 2018;52(4):1893-907. doi: 10.1007/s11135-017-0574-8 [published Online First: 2017/09/14]

83. Hennink MM, Kaiser BN, Marconi VC. Code Saturation Versus Meaning Saturation: How Many Interviews Are Enough? *Qualitative Health Research* 2016;27(4):591-608. doi: 10.1177/1049732316665344

For peer review only

84. Masculinities Project [Available from: https://www.masculinitiesproject.org/ accessed 29.06.2020 2020.
85. Timmermans S, Tavory I. Theory Construction in Qualitative Research From Grounded Theory to Abductive Analysis. *Sociological Theory* 2012;30:167-86. doi: 10.1177/0735275112457914
86. What is RQDA and what are its features? [Available from: http://rqda.r-forge.r-project.org/ accessed 28.06.2020 2020.
87. Dillon B. Using mobile phones to collect panel data in developing countries. *Journal of International Development* 2012;24(4):518-27. doi: 10.1002/jid.1771

For peer review only

For peer review only - http://bmjopen.bmj.com/site/about/guidelines.xhtml
